# Epigenetic Regulation of CD8^+^ Effector T Cell Differentiation by PDCD5

**DOI:** 10.1002/eji.202451388

**Published:** 2025-03-20

**Authors:** Lixue Jin, Xin Zhang, Jingyi Wang, Yujia Wang, Ke Wang, Zhuolin Wang, Pingzhang Wang, Xiuyuan Sun, Jie Hao, Rong Jin, Dan Lu, Qing Ge

**Affiliations:** ^1^ Department of Immunology, School of Basic Medical Sciences, NHC Key Laboratory of Medical Immunology, Beijing Key Laboratory of Tumor Systems Biology, Institute of Systems Biomedicine, Peking University Health Science Center Peking University Beijing China; ^2^ Department of Immunology School of Basic Medical Sciences NHC Key Laboratory of Medical Immunology Beijing Key Laboratory of Tumor Systems Biology Institute of Systems Biomedicine Peking University Health Science Center Beijing China; ^3^ Department of Integration of Chinese and Western Medicine School of Basic Medical Sciences Peking University Beijing China

**Keywords:** CD8^+^ T cells, effector T cell differentiation, epigenetic modification, LCMV Clone 13 virus, PDCD5

## Abstract

Epigenetic modification plays a crucial role in establishing the transcriptional program that governs the differentiation of CD8^+^ effector T cells. However, the mechanisms by which this process is regulated at an early stage, prior to the expression of master transcription factors, are not yet fully understood. In this study, we have identified PDCD5 as an activation‐induced molecule that is necessary for the proper differentiation and expansion of antigen‐specific CD8^+^ effector T cells in a mouse model of chronic viral infection. The genetic deletion of *Pdcd5* resulted in impaired differentiation and function of effector T cells, while T‐cell activation, metabolic reprogramming, and the differentiation of memory/exhausted T cells were largely unaffected. At the molecular level, we observed reduced chromatin accessibility and transcriptional activity of *Tbx21* and its regulated genes in *Pdcd5* ^−/−^ CD8^+^ T cells. We further identified that PRDM9 facilitates the H3K4me3 modification of genes associated with the effector phenotype in CD8^+^ T cells. The interaction between PDCD5 and PRDM9 promotes the nuclear translocation and lysine methyltransferase activity of PRDM9. Collectively, these findings highlight the crucial role of the PDCD5/PRDM9 axis in epigenetic reprogramming during the early stages of fate determination for effector CD8^+^ T cell fate.

## Graphical abstract

In CD8^+^ T cells, PDCD5 is an activation‐induced molecule that interacts with PRDM9, thereby promoting its nuclear translocation and facilitating the H3K4me3 modification of genes associated with effector differentiation.

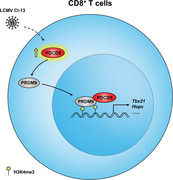

## 1 Introduction

T‐cell recognition of specific peptide‐MHC complexes triggers a cascade of biochemical, metabolic, and genetic events, leading to proliferation approximately 24 h later [1]. CD8^+^ T cells, particularly those undergoing cell division, function as bipotent intermediates that dynamically modulate the epigenetic regulation of either effector or memory programs. This process determines the transcriptional state of lineage‐specifying genes [2]. For example, histone methyltransferases EZH2 and Suv39h1, as well as the histone demethylase Utx, repress the accessibility of promemory genes during relatively late phases to facilitate the development of terminally differentiated CD8^+^ effector T cells [3, 4, 5]. Conversely, the histone deacetylase HDAC3 suppresses the expression of pro‐effector genes early during CD8^+^ effector T cell commitment [6]. Moreover, many lineage‐specifying transcription factors, including *Tbx21*—which is crucial for the differentiation of CD8^+^ effector and CD4^+^ T helper 1 cells—exhibit a “bivalent” gene state. These genes possess both activating H3K4me3 and repressing H3K27me3 marks at their regulatory regions [7, 8], enabling dynamic regulation of these master transcription factors. However, the mechanisms governing the precise regulation of chromatin accessibility patterns in relation to effector and memory differentiation are still not fully understood.

Programmed cell death 5 (PDCD5) is a small protein expressed in various tissues and involved in a range of physiological and pathological processes [9]. Its functions include inducing apoptosis by enhancing the stability and activation of p53 in tumor cells [9, 10], influencing vascular remodeling by reducing nitric oxide production in endothelial cells [11], and promoting lung fibrosis by facilitating the expression of matricellular genes induced by TGF‐β in club cells [12]. Although the roles of PDCD5 in T cells are not well understood, some data suggest that PDCD5 may influence the fate of T cell subsets by regulating the expression or activity of transcription factors that specify lineage identity. For instance, the overexpression of PDCD5 enhances the acetylation and activity of Foxp3, promoting the differentiation of regulatory T (Treg) cells and providing protection against experimentally induced autoimmune encephalomyelitis in mice [13]. PDCD5 also upregulates the expression of T‐bet in invariant natural killer T (iNKT) cells, facilitating the differentiation of NKT1 cells that produce IFN‐γ [14]. However, it remains unclear whether PDCD5 regulates the differentiation of other T cell subsets, particularly CD8^+^ T cells.

In this study, we employed mice with T‐cell‐specific deletion of *Pdcd5* to explore its role in the differentiation of CD8^+^ T cells during viral infection. Our findings demonstrate that following T‐cell activation, upregulation of PDCD5 and its translocation to the nucleus contribute to the epigenetic programming of T cells, ultimately influencing their terminal differentiation into functional effector cells.

## 2 Results

### 2.1 Pdcd5‐Deficient Mice Have an Impaired Antiviral Response Upon LCMV Cl‐13 Infection

**FIGURE 1:**
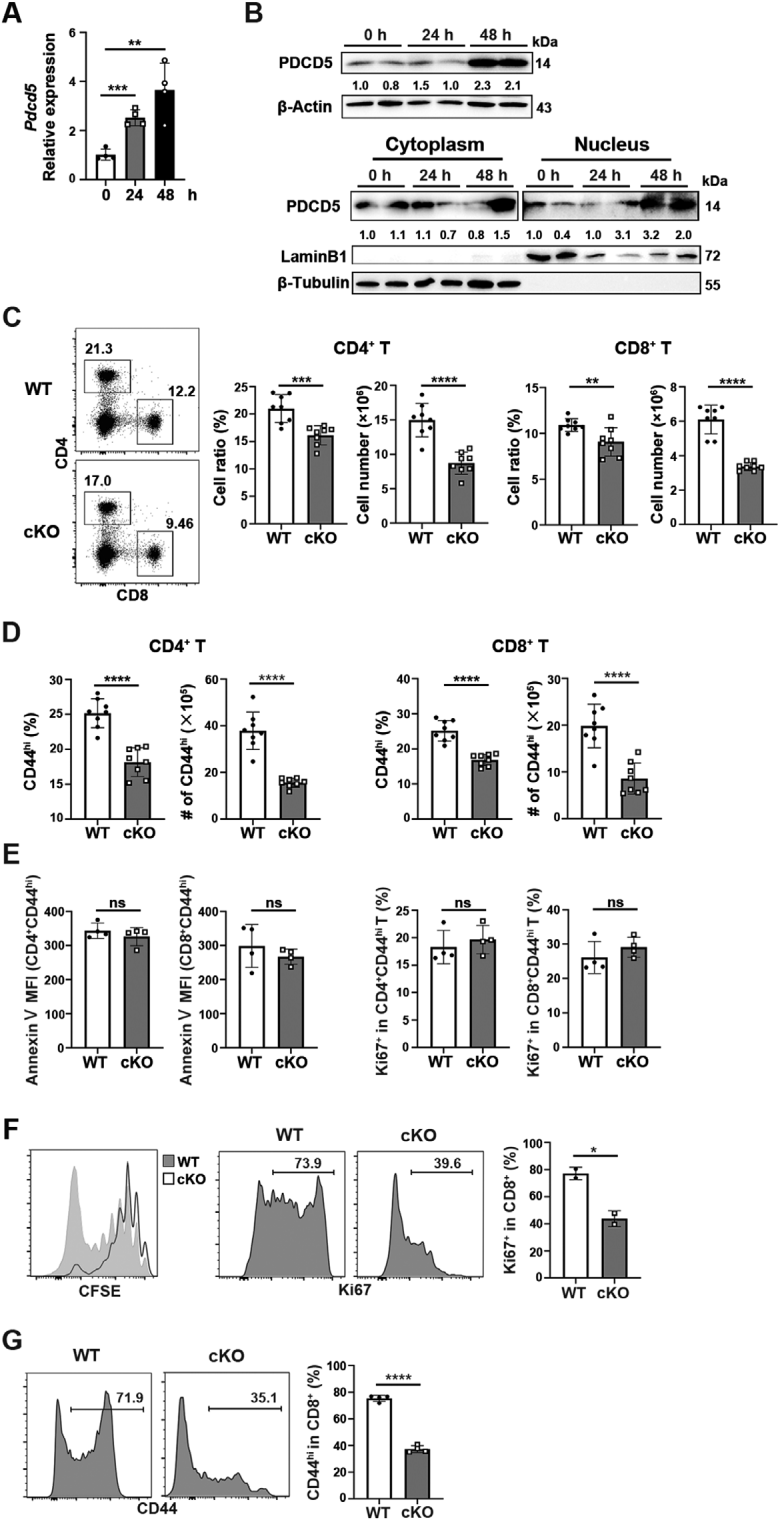
T cells upregulate PDCD5 upon activation, and deletion of *Pdcd5* in T cells results in reduced CD44^hi^ peripheral T cells. (A) Quantitative RT‐PCR analysis of *Pdcd5* in purified CD44^lo^CD62L^hi^ murine naïve CD8^+^ T cells upon stimulation with anti‐CD3 and anti‐CD28 antibodies. β‐actin was used as the housekeeping gene control. (B) Western blotting of total PDCD5 (upper panel) or cytoplasmic/nucleic PDCD5 (lower panel) in purified naïve CD8^+^ T cells upon stimulation with anti‐CD3 and anti‐CD28 antibodies. (C, D) Flow cytometry analysis of total (C) and CD44^hi^ (D) CD4^+^ and CD8^+^ T cells in the spleen of *Pdcd5* ^fl/fl^ (WT) and *Pdcd5* ^fl/fl^ *Cd4*‐Cre (cKO) mice. The percentages and numbers of total and indicated T cell subsets were calculated. (E) Comparison of the mean fluorescence intensity (MFI) of Annexin V staining (left panel) and percentage of Ki67^+^ (right panel) cells in CD4^+^CD44^hi^ and CD8^+^CD44^hi^ splenic T cells between WT and cKO mice. (F, G) Flow cytometry analysis of T cell proliferation (F) and CD44 upregulation (G) in RAG^−/−^ mice. Purified CD8^+^CD44^lo^CD62L^hi^CD25^−^ T cells obtained from *Pdcd5* ^fl/fl^ (WT) and *Pdcd5* ^fl/fl^ *Cd4*‐Cre (cKO) mice were labeled with CFSE and were adoptively transferred into RAG1^−/−^ mice. The cell proliferation was examined by CFSE dilution (left) and Ki67 staining (right). Representative flow cytometry plots were shown, and the percentage of Ki67^+^ or CD44^hi^ cells in CD8^+^ T cells were calculated. The experiments were repeated 2–3 times. The student's *t*‐test was used for statistical analysis. Mean ± standard deviation (SD), * *p* < 0.05, ** *p* < 0.01, *** *p* < 0.001, **** *p* < 0.0001, ns, not significant.

To investigate the functions of PDCD5 in CD8^+^ T cell differentiation, we first examined whether PDCD5 expression is upregulated during T‐cell activation. We observed a significant increase in both mRNA and protein levels of PDCD5 in CD8^+^ T cells 48 h after stimulation with anti‐CD3 and anti‐CD28 antibodies (Figure 1A,B). Nuclear translocation of PDCD5 was also detected at this time point (Figure 1B). To further support our findings, we analyzed publicly available single‐cell RNA sequencing (scRNA‐seq) data from healthy donors and patients with cancers or COVID‐19 infection (GSE164522, GSE156728, GSE155223) [15, 16, 17]. The analysis revealed a high level of *PDCD5* transcription in proliferating CD8^+^ and CD4^+^ T cells (Figure S1A–C).

Next, we examined T cells in mice with T‐cell‐specific deletion of Pdcd5 (*Pdcd5* ^fl/fl^ *Cd4*‐Cre or cKO mice) (Figure S1D–E) under steady‐state conditions. T‐cell development in the thymus appeared normal in cKO mice (Figure S1F). However, the numbers of T cells in the spleen, as well as the frequencies and numbers of CD44^hi^ antigen‐experienced T cells in the lymph nodes and spleen, were reduced in cKO mice compared with *Pdcd5* ^fl/fl^ *Cd4*‐Cre^−^ (WT) littermate controls (Figure 1C,D; Figure S1E). Notably, the reduction of CD44^hi^ cells in the lymph nodes was more pronounced in CD8^+^ T cells (Figure S1G). Under steady‐state conditions, the proliferation and apoptosis rates of CD44^hi^ T cells were similar between WT and cKO mice (Figure 1E). When purified naïve CD8^+^ T cells (CD44^lo^CD62L^hi^CD25^−^) were adoptively transferred into lymphopenic RAG1^−/−^ mice, a defect in T cell proliferation and CD44 upregulation was observed in *Pdcd5* ^−/−^ T cells 6 days later (Figure 1F,G). Together, these results suggest that PDCD5 may be involved in regulating T‐cell activation and/or differentiation at an early stage.

**FIGURE 2:**
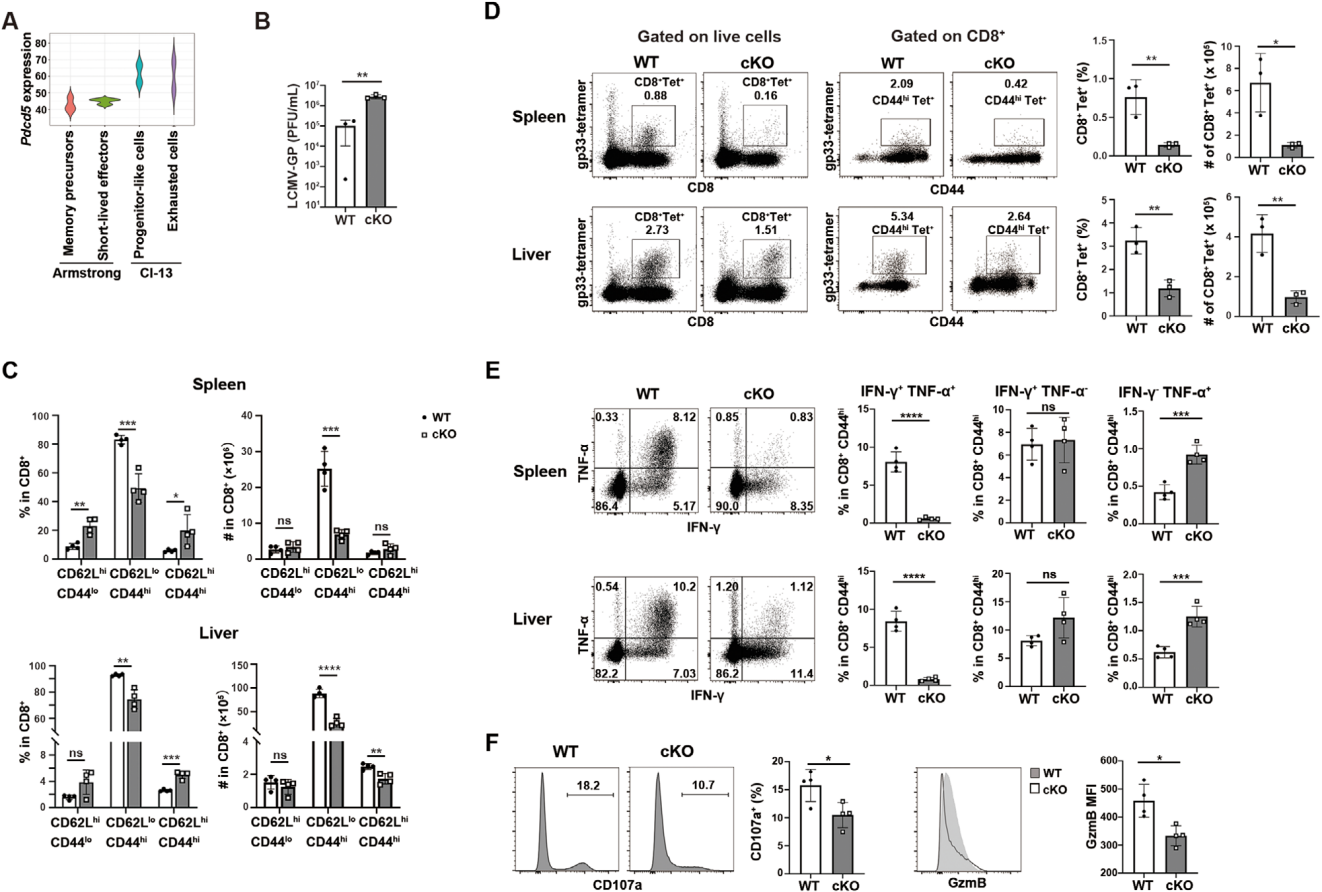
*Pdcd5*‐deficient CD8^+^ T cells have impaired antiviral responses. WT and cKO mice were infected with LCMV Cl‐13 at 4 × 10^5^ plaque‐forming units (PFU) via intraperitoneal (i.p.) injection. (A) Transcriptome comparison of *Pdcd5* expression in P14 T cells at 7 days post LCMV infection. The bulk RNA sequencing data of P14 transgenic T cells at 7 days post LCMV Armstrong or Cl‐13 infection with the accession number GSE119943 were analyzed. (B) Quantitative RT‐PCR of LCMV‐glycoprotein (GP) mRNA in peripheral blood at 10 days postinfection (dpi). The mRNA of a series of dilutions of stock LCMV with known viral titers was used as a standard curve for calculating the viral titer in peripheral blood samples. (C) Flow cytometry analysis of the percentages and cell numbers of CD44^hi^CD62L^lo^, CD44^hi^CD62L^hi^, CD44^lo^CD62L^hi^ cells in CD8^+^ T cells in the spleen and liver of WT and cKO mice at 10 dpi. (D) Flow cytometry analysis of the percentages and cell numbers of CD8^+^ gp33‐tetramer (Tet)^+^ cells in the spleen and liver of WT and cKO mice at 10 dpi. The CD44 expression in CD8^+^Tet^+^ cells was also shown. (E) Intracellular staining of IFN‐γ and TNF‐α in splenic and liver CD8^+^CD44^hi^ cells collected at 10 dpi following 4 h of GP_33‐41_ peptide stimulation and BFA (3 µg/mL) blockade. The percentages of IFN‐γ^+^TNF‐α^+^, IFN‐γ^+^TNF‐α^−^, IFN‐γ^−^TNF‐α^+^ cells are shown. (F) Comparison of the percentage of CD107a^+^ (top panel) and MFI of Granzyme B (GzmB, lower panel) in splenic CD8^+^CD44^hi^ cells collected at 10 dpi following 4 h of GP_33‐41_ peptide stimulation. The experiments were repeated more than three times, and representative data are shown. The student's *t*‐test was used for statistical analysis. Mean ± SD, * *p* < 0.05, ** *p* < 0.01, *** *p* < 0.001, **** *p* < 0.0001, ns, not significant.

We then investigated the impact of PDCD5 on CD8^+^ T cell activation/differentiation using a lymphocytic choriomeningitis virus (LCMV) infection mouse model. As shown in Figure 2A, the expression levels of *Pdcd5* were higher in the public available transcriptome of P14 TCR transgenic T cells following chronic infection with the Clone‐13 (Cl‐13) strain compared with acute infection with the Armstrong strain at 7 days postinfection (dpi) (GSE119943) (Figure 2A) [18]. Based on these findings, we infected WT and *Pdcd5* cKO mice with LCMV Cl‐13 [19]. At 10 dpi, cKO mice had higher viral titers in plasma compared with WT mice (Figure 2B). The frequencies and numbers of CD44^hi^CD62L^lo^CD8^+^ cells and gp33‐tetramer (Tet)^+^CD8^+^ T cells were decreased in the spleen and liver of cKO mice at 10 dpi (Figure 2C,D). Following in vitro stimulation with LCMV GP_33‐41_ peptide, splenic and liver CD8^+^ T cells from cKO mice exhibited significantly lower percentages of IFN‐γ^+^TNF‐α^+^ cells and CD107a^+^ cells, as well as reduced levels of granzyme B compared with WT mice (Figure 2E,F). As T cells producing both IFN‐γ and TNF‐α are proved to have higher levels of cytotoxicity and mediate more efficient pathogen killing compared with those that produce either cytokine alone [20], these results suggest that the antiviral response of *Pdcd5^−/−^* CD8^+^ T cells is impaired at the early stages of infection.

We also assessed the antiviral responses of CD8^+^ T cells 30 days post intraperitoneal injection of LCMV Cl‐13 virus. Higher viral titers were observed in the liver, lung, brain, and kidney of cKO mice compared with WT ones (Figure S2A). At this time point, *Pdcd5* ^−/−^ CD8^+^ T cells exhibited reduced numbers of CD44^hi^CD62L^lo^ cells in the spleen and higher expression of inhibitory molecules PD‐1 and TIM‐3 in CD44^hi^ cells (Figure S2B,C). Similar results were found when the mice were infected with LCMV Cl‐13 by intravenous injection (Figure S2D–F). Collectively, these data suggest that PDCD5 plays a crucial role in promoting antiviral responses.

### 2.2 Pdcd5‐Deficient Mice Have Reduced Effector CD8^+^ T Cells Early After LCMV Cl‐13 Infection

**FIGURE 3:**
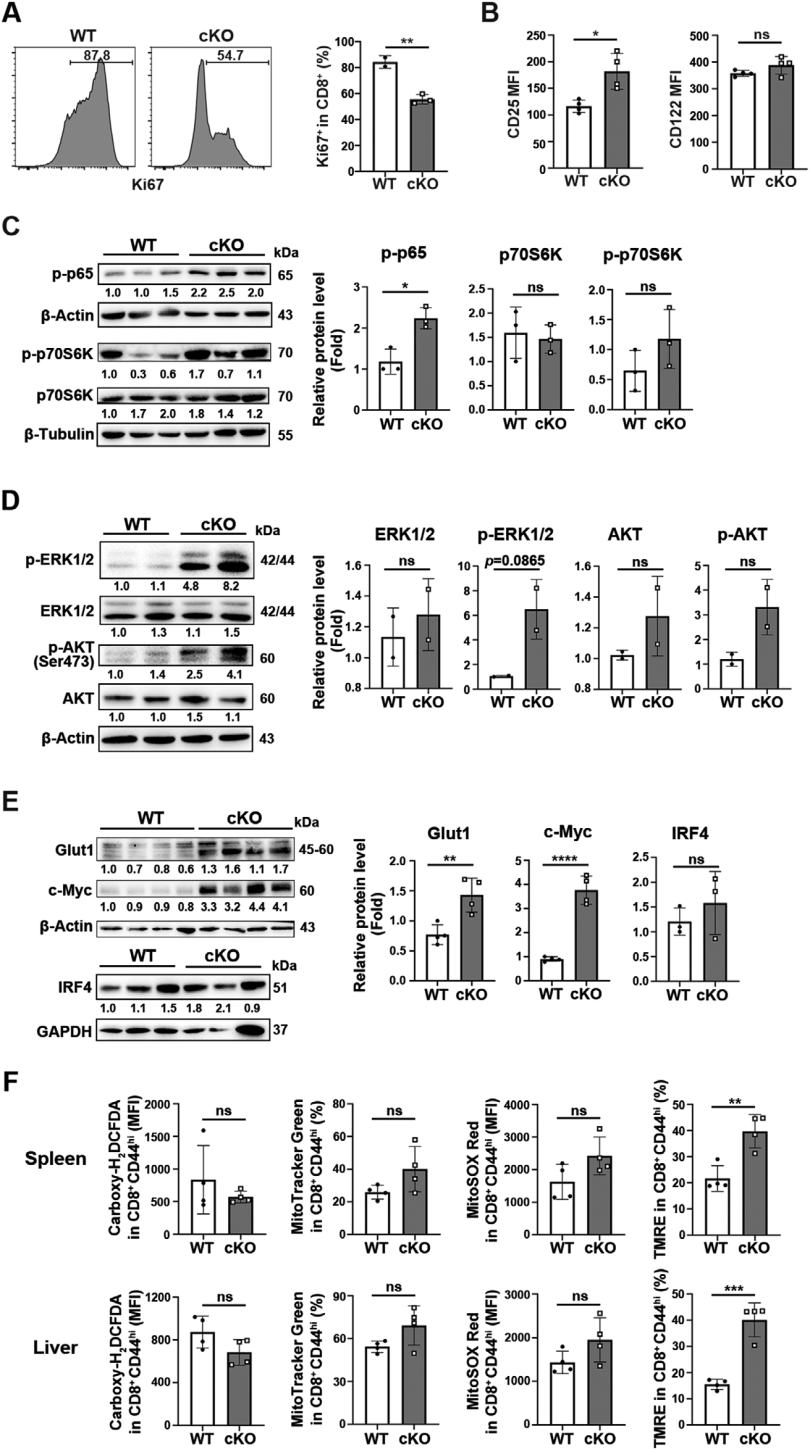
Reduced proliferation but not activation in *Pdcd5*‐deficient CD8^+^ T cells upon LCMV Cl‐13 infection. WT and cKO mice were infected with LCMV Cl‐13 at 4 × 10^5^ PFU via intraperitoneal injection. The splenic cells were harvested at 10 dpi for either flow cytometry analysis or magnetic bead‐based CD8^+^ T cell sorting. (A) Flow cytometry analysis of the percentages of Ki67^+^ cells in WT and *Pdcd5* ^−/−^ CD8^+^ T cells. (B) Comparison of the MFI of CD25 and CD122 expression in WT and *Pdcd5* ^−/−^ CD8^+^ T cells. (C, D) Immunoblot analysis of phosphorylated p65, p70S6K, ERK1/2, and AKT in purified WT and *Pdcd5* ^−/−^ CD8^+^ T cells. Gray values of the indicated proteins were determined for statistical analysis on the right. (E) Western blotting of Glut1, c‐Myc, and IRF4 in purified WT and *Pdcd5* ^−/−^ CD8^+^ T cells. Gray values of the indicated proteins were determined for statistical analysis on the right. (F) Flow cytometry analysis of cellular reactive oxygen species (ROS, carboxy‐H_2_DCFDA staining), mitochondrial content (MitoTracker Green staining), mitochondrial ROS (MitoSOX Red staining), and mitochondrial membrane potential (TMRE staining) in WT and *Pdcd5* ^−/−^ CD8^+^ T cells. Data are representative of 2–3 independent experiments. The student's *t*‐test was used for statistical analysis. Mean ± SD, * *p* < 0.05, ** *p* < 0.01, *** *p* < 0.005, ns, not significant.

We next examined the effects of *Pdcd5* deficiency on virus‐specific T cell proliferation and effector T (Teff) cell differentiation during LCMV Cl‐13 infection. Compared with WT mice, cKO mice exhibited a significantly lower percentage of Ki67^+^ CD8^+^ T cells at 10 dpi (Figure 3A), indicating impaired T cell expansion. We also assessed the expression of IL‐2 and IL‐15 receptors (CD25 and CD122, respectively) on *Pdcd5* ^−/−^ T cells and found that CD25 levels were enhanced, while CD122 levels were unchanged compared with WT T cells (Figure 3B). To evaluate T cell activation, we measured the activation of NF‐κB, MAPK, and PI3K/AKT signaling pathways. At 10 dpi, *Pdcd5* ^−/−^ CD8^+^ T cells exhibited higher levels of phosphorylated p65 (a subunit of NF‐κB), while the phosphorylation of ERK, AKT, and p70S6K was comparable between the two groups (Figure 3C,D). Additionally, we observed a significant increase in the expression of Glut1 and c‐Myc [21] in *Pdcd5* ^−/−^ T cells (Figure 3E). We further examined reactive oxygen species (ROS) levels, mitochondrial content, mitochondrial ROS, and mitochondrial membrane potential in the CD44^hi^ subpopulation of both WT and cKO mice. No significant differences were observed except for an increase in mitochondrial membrane potential in *Pdcd5* ^−/−^ T cells (Figure 3F). These data suggest that the impaired expansion of *Pdcd5* ^−/−^ CD8^+^ T cells after LCMV infection cannot be attributed to altered T cell activation or mitochondrial function.

**FIGURE 4:**
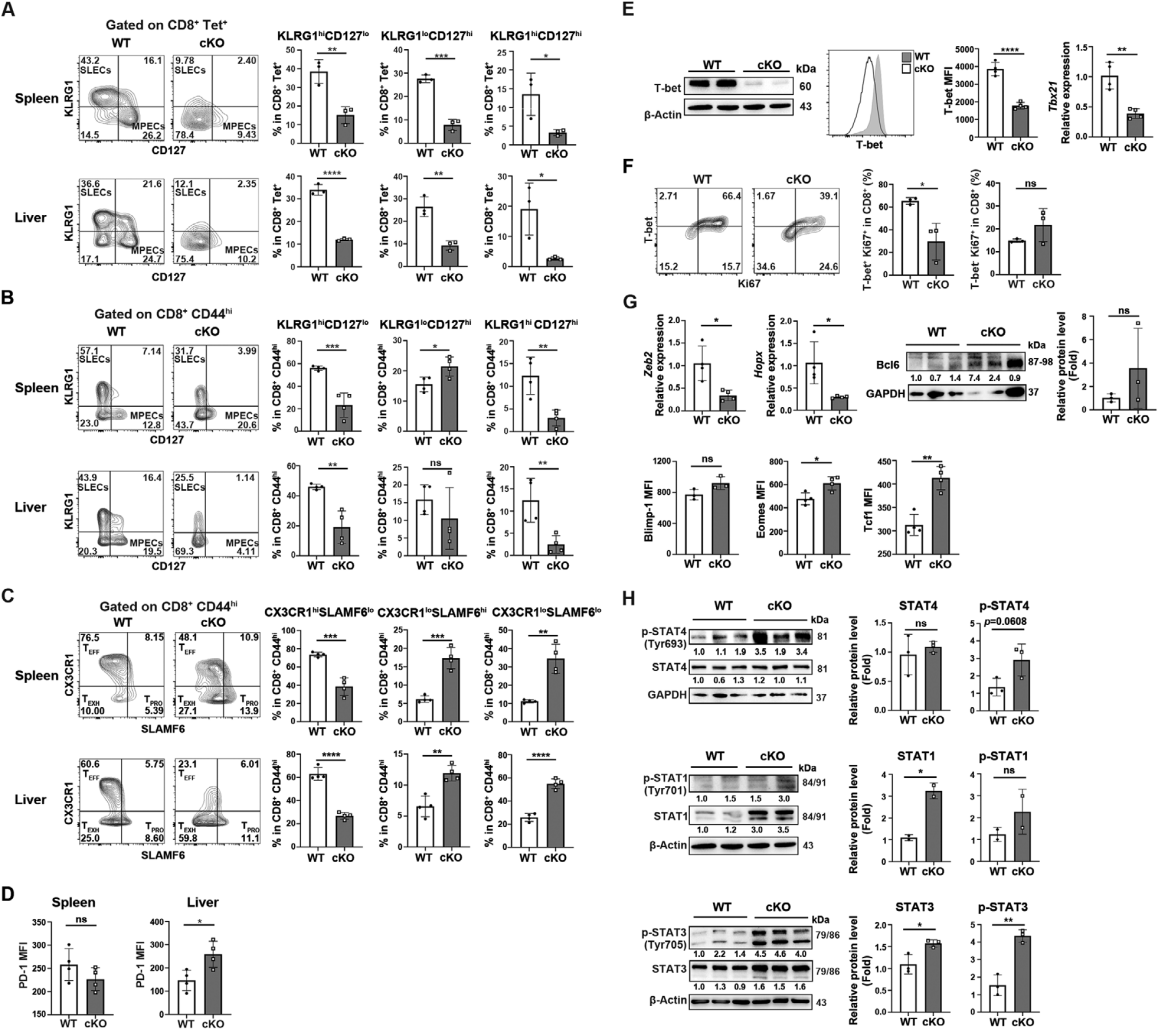
Impaired CD8^+^ effector T cell differentiation in *Pdcd5*‐deficient mice upon LCMV Cl‐13 infection. WT and cKO mice were infected with LCMV Cl‐13 at 4×10^5^ PFU via intraperitoneal injection. The splenic cells were harvested at 10 dpi for either flow cytometry analysis or magnetic bead‐based CD8^+^ T cell sorting (for western blotting or RT‐PCR analysis). (A, B) Flow cytometry analysis of KLRG1 and CD127 expression in WT and *Pdcd5* ^−/−^ CD8^+^Tet^+^ (A) or CD8^+^CD44^hi^ (B) T cells. The percentages of indicated T cell subsets were calculated for statistical analysis and are shown on the right. (C) Flow cytometry comparison of CX3CR1 and SLAMF6 expression in WT and *Pdcd5* ^−/−^ CD8^+^CD44^hi^ T cells. The percentages of indicated T cell subsets were calculated for statistical analysis and are shown on the right. (D) Flow cytometry analysis of PD‐1 expression in splenic and liver CD8^+^CD44^hi^ T cells. The mean fluorescence intensities (MFI) of PD‐1 were calculated for statistical analysis. (E) Comparison of T‐bet protein expression measured by immunoblot (left) and flow cytometry (middle) and *Tbx21* mRNA level measured by quantitative RT‐PCR (right). (F) Comparison of the percentages of T‐bet^+^Ki67^+^ and T‐bet^−^Ki67^+^ CD8^+^ T cells between WT and cKO mice at 10 dpi. (G) Comparison of *Zeb2* and *Hopx* transcription (quantitative RT‐PCR), Bcl‐6 (immunoblotting), Blimp‐1, Eomes, and Tcf1 (flow cytometry) protein levels in splenic CD8^+^ T cells between WT and cKO mice. (H) Western blotting analysis of total and phosphorylated STAT4, STAT1, and STAT3 in CD8^+^ T cells at 10 dpi. Gray values of the indicated proteins were determined for statistical analysis on the right. The experiments were repeated for 2–3 times, and representative data are shown. The student's *t*‐test was used for statistical analysis. Mean ± SD, * *p* < 0.05, ** *p* < 0.01, *** *p* < 0.001, **** *p* < 0.0001, ns, not significant.

We then investigated the effect of *Pdcd5* deficiency on Teff cell differentiation. At 10 dpi, cKO mice exhibited significant reductions in the percentages of CX3CR1^hi^SLAMF6^lo^ and CD127^lo^KLRG1^hi^ cells, which are hallmarks of Teff cells (Figure 4A–C). However, the percentages of T cells with pre‐exhausted progenitor (CX3CR1^lo^SLAMF6^hi^) or memory precursor phenotype (CD127^hi^KLRG1^lo^) were not consistently decreased in cKO mice (Figure 4A–C). We also assessed T cell exhaustion by measuring PD‐1 expression and found increased PD‐1 expression in *Pdcd5* ^−/‐^ T cells in the liver but not the spleen (Figure 4D).

To further explore the molecular mechanisms underlying impaired Teff cell differentiation in *Pdcd5* ^−/−^ CD8^+^ T cells, we examined the expression of critical transcription factors involved in Teff cell differentiation, including T‐bet (encoded by *Tbx21*), Blimp1, Zeb2, and Hopx [22, 23, 24, 25, 26, 27]. Compared with WT CD8^+^ T cells, *Pdcd5* ^−/−^ cells exhibited significant decreases in the protein level of T‐bet and mRNA levels of *Tbx21*, *Zeb2*, and *Hopx* at 10 dpi (Figure 4E–G). The prevalence of T‐bet^+^Ki67^+^ CD8^+^ T cells was also decreased in cKO mice, while the prevalence of T‐bet^−^Ki67^+^ remained unchanged (Figure 4F). In contrast, the expression of transcription factors associated with memory differentiation, such as Eomes, Tcf1, and Bcl6 [28, 29, 30], was either increased or comparable in *Pdcd5* ^−/−^ CD8^+^ T cells compared with WT cells (Figure 4G). We also examined the activation patterns of STAT4, STAT1, and STAT3, which are involved in cytokine signaling pathways. These pathways are known to influence Teff cell differentiation through cytokines such as IL‐12 and type I interferon (IFN), which promote Tcf1 downregulation and Teff cell differentiation, while IL‐21 and IL‐10 facilitate memory cell formation [31, 32, 33]. However, the activation patterns of these STAT proteins did not explain the defective Teff cell formation in Pdcd5^−/−^ CD8^+^ T cells (Figure 4H). Collectively, these results indicate that *Pdcd5* deficiency impairs the effector differentiation program in CD8^+^ T cells during LCMV Cl‐13 infection, while their cytokine responsiveness remains unaffected.

### 2.3 PDCD5 is Required in a Cell‐Autonomous Manner for CD8^+^ Teff Cell Differentiation

**FIGURE 5:**
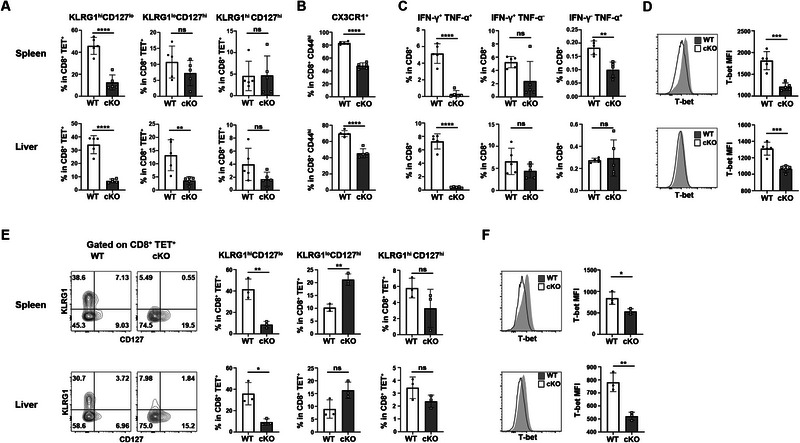
Cell autonomous regulation of CD8^+^ Teff cell differentiation by PDCD5. (A–D) Analysis of *Pdcd5* ^−/−^ CD8^+^ Teff cell differentiation in CD4‐depleted mouse model. CD4‐specific antibody (200 µg/mouse) was intraperitoneally injected into WT and cKO mice 1 day prior and 1 day after LCMV Cl‐13 infection. The spleen and liver were harvested at 10 dpi for flow cytometry analysis. The percentages of indicated T cell subsets in CD8^+^Tet^+^ cells are shown in (A), and those in CD8^+^CD44^hi^ cells are shown in (B). The T cells were restimulated in vitro by GP_33‐41_ peptide, and the percentages of IFN‐γ^+^TNF‐α^+^, IFN‐γ^+^TNF‐α^−^, IFN‐γ^−^TNF‐α^+^ in CD8^+^ T cells were measured by flow cytometry (C). The MFI of T‐bet expression between WT and *Pdcd5* ^−/−^ CD8^+^ T cells were compared in (D). (E, F) Analysis of *Pdcd5* ^−/−^ CD8^+^ Teff cell differentiation in mixed bone marrow (BM) chimeric mouse model. Mixed BM chimeric mice were generated by coinjecting cKO CD45.2^+^ and WT CD45.1^+^ BM cells (1:1 ratio) into lethally irradiated CD45.1^+^ WT mice. The chimeric mice were infected with LCMV Cl‐13 at 7 weeks after reconstitution. Ten days later, the percentages of indicated T cell subsets (E) and the expression of T‐bet (F) in CD8^+^ Tet^+^ T cells were analyzed by flow cytometry. Data are representative of two independent experiments. The student's *t*‐test was used for statistical analysis. Mean ± SD, * *p* < 0.05, ** *p* < 0.01, *** *p* < 0.001, **** *p* < 0.0001, ns, not significant.

CD4^+^ T cell help is crucial for the differentiation of CD8^+^ Teff cells during chronic infection [34]. To exclude the potential impact of defective CD4^+^ T cell help in cKO mice, we depleted CD4^+^ T cells using a CD4‐specific antibody prior to LCMV Cl‐13 infection (Figure S3A). Despite this depletion, cKO mice exhibited similarly less weight loss around 7 dpi (Figure S3B). At 10 dpi, we observed similarly reduced total CD8^+^ T cell numbers, decreased frequencies of CD127^lo^KLRG1^hi^ in CD8^+^Tet^+^ cells, decreased proportion of CD44^hi^CD62L^lo^, CX3CR1^+^, IFN‐γ^+^TNF‐α^+^ CD8^+^Teff cells, as well as reduced T‐bet expression in the spleen and liver of cKO mice compared with WT mice (Figure 5A–D; Figure S3C,D).

To further investigate whether these defects were intrinsic to CD8^+^ T cells, we generated 50:50 mixed bone marrow (BM) chimeras using cKO CD45.2^+^ and WT CD45.1^+^ progenitor cells. After reconstitution, these chimeric mice were infected with LCMV Cl‐13. Consistent with our findings in cKO mice, we observed reduced percentages of total CD8^+^ and CD8^+^Tet^+^ cells, decreased frequencies of CD127^lo^KLRG1^hi^ CD8^+^Tet^+^ cells, as well as the decreased ratio of CD44^hi^CD62L^lo^ and Ki67^+^ CD8^+^ cells. Additionally, T‐bet expression was also decreased in the CD45.2^+^ *Pdcd5* ^−/−^ CD8^+^ T cell compartment (Figure 5E,F; Figure S3E–G). However, the percentages of KLRG1^lo^CD127^hi^ memory precursor cells were not consistently altered in either model (Figure 5A,E). These results collectively indicate that the defects in Teff differentiation in cKO mice are intrinsic to CD8^+^ T cells and independent of CD4^+^ T cell help.

### 2.4 Transcriptome of Pdcd5‐Deficient CD8^+^ T Cells Exhibit Defects in Effector T Cell Differentiation

**FIGURE 6:**
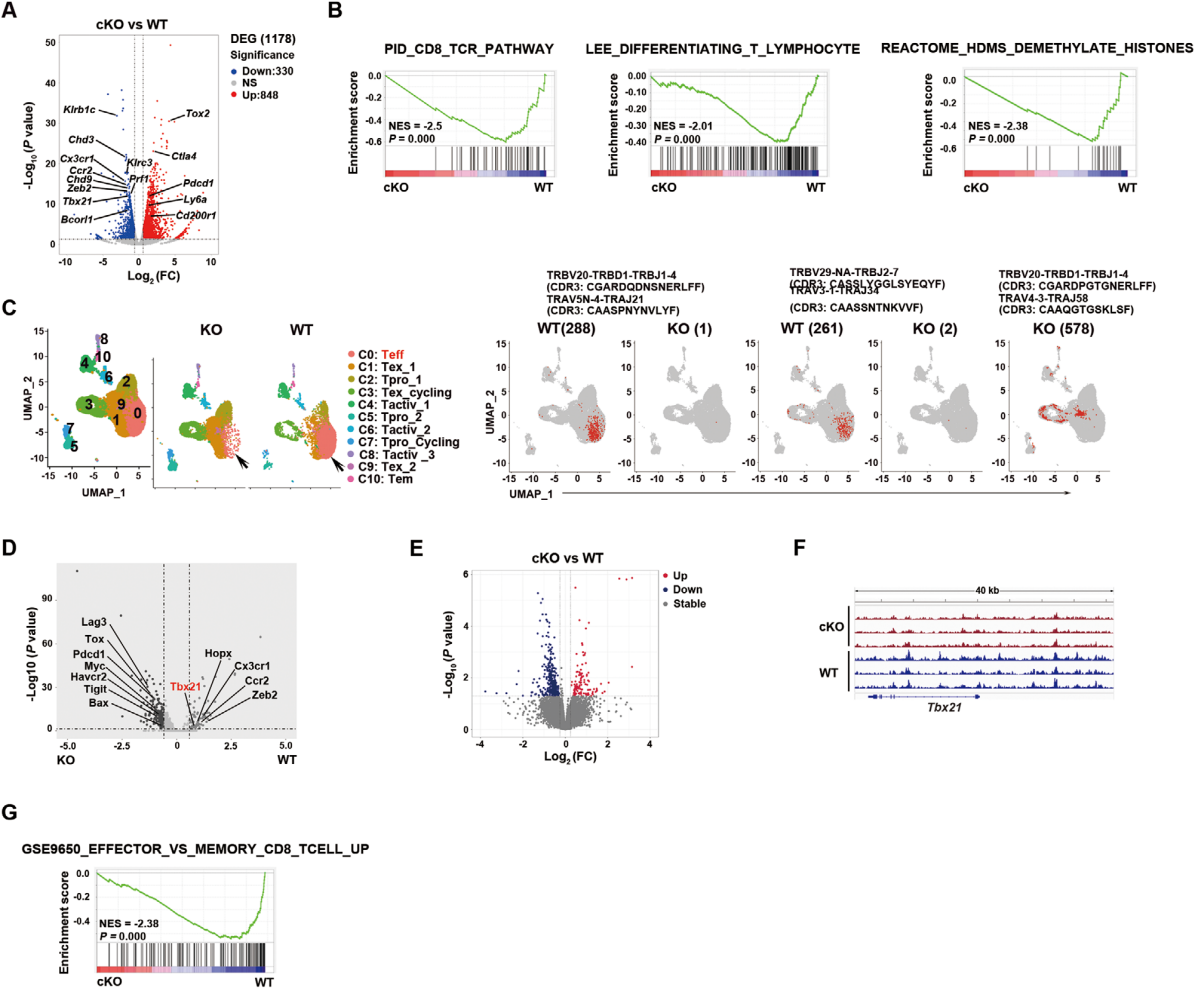
Reduced transcription and chromatin accessibility of effector signature genes in *Pdcd5* ^−/−^ CD8^+^ T cells 10 days after LCMV Cl‐13 infection. WT and cKO mice were infected with LCMV Cl‐13 at 4 × 10^5^ PFU via intraperitoneal injection. Ten days later, the splenic CD8^+^ T cells were purified by magnetic bead‐based sorting and were subjected to bulk RNA sequencing (two mice in each group, A, B), scRNA‐seq (2 mice in each group, C, D), and ATAC sequencing (three mice in each group, E–G). (A) Volcano plot showing differentially expressed genes (DEGs) in WT and *Pdcd5* ^−/−^ CD8^+^ T cells (bulk RNA‐seq). The dotted lines show a two‐fold cutoff. (B) GSEA analysis of DEGs between WT and *Pdcd5* ^−/−^ CD8^+^ T cells (bulk RNA‐seq). NES represents the normalized enrichment score. (C) UMAP (left) visualization of CD8^+^ T cell clusters and UMAP plots (right) with red dots displaying indicated TCR clones (gray for all other T cells, scRNA‐seq). The cell numbers of each clone within WT or *Pdcd5* ^−/−^ CD8^+^ T cells are indicated in the parentheses. (D) Volcano plot comparing differentially expressed genes between cKO and WT CD8^+^ T cells obtained from the most expanded TCR clone (scRNA‐seq). The dotted lines show a 1.5‐fold cutoff. (E) Volcano plot analysis of pairwise comparison of ATAC‐seq density between WT and *Pdcd5* ^−/−^ CD8^+^ T cells. The dotted lines show a two‐fold cutoff. (F) Integrative genomics viewer (IGV) analysis of ATAC‐seq coverage of *Tbx21*. (G) GSEA of the differentially accessible gene regions in WT and *Pdcd5* ^−/−^ CD8^+^ T cells (ATAC‐seq).

To better understand the defective Teff cell differentiation in *Pdcd5*‐deficient T cells, we performed bulk RNA‐seq and scRNA‐seq. CD8^+^ splenic T cells purified from LCMV Cl‐13‐infected WT and cKO mice at 10 dpi were compared. Consistent with the observation of fewer Teff cells in cKO mice, the bulk RNA‐seq of *Pdcd5* ^−/−^ T cells exhibited decreased expression levels of Teff or short‐lived effector cell signature genes [23], with a particularly notable down‐regulation of *Tbx21* and its target genes (Figure 6A; Figure S4A). Conversely, the transcription of several inhibitory molecules, such as *Pdcd1* and *Ctla4*, was increased in *Pdcd5* ^−/−^ T cells (Figure S4A). No significant differences were observed in the transcription of memory T cell‐related transcription factors such as *Bcl6*, *Tcf1*, and *Eomes* between the two groups (data not shown). KEGG pathway analysis revealed that down‐regulated genes were enriched for natural killer cell‐mediated cytotoxicity (*p* = 0.0001107), while GSEA analysis showed enrichment for the CD8_TCR_pathway, Lee_differentiating_T_lymphocytes, HDMS_demethylate_histones (Figure 6B; Figure S4B)

We also analyzed the scRNA‐seq results of WT and *Pdcd5* ^−/−^ CD8^+^ T cells and identified 11 distinct cell clusters. These included one Teff cell cluster (C0), characterized by *Tbx21* ^hi^ *Zeb2* ^hi^ *Cxcr6* ^hi^; two exhausted T cell (Tex) clusters (C1 and C9), marked by *Pdcd1* ^+^ *Tigit* ^+^ *Tox* ^+^; two resting and/or pre‐exhausted progenitor (Tpro) clusters (C2 and C5) identified by *Id3* ^+^ *Slamf6* ^+^; one cycling cluster (C3) with a Tex signature; and one cycling cluster (C5) with a progenitor‐like signature (Figure 6C; Figure S4C–E). Notably, *Pdcd5* expression was high in approximately half of the Teff (C0) and the majority of Tex_1 (C1) cluster cells (Figure S4F).

Compared with WT T cells, *Pdcd5* ^−/−^ T cells exhibited a significant reduction in the number of cells within the Teff cluster (C0) (Figure S4G). To further explore this, we performed single‐cell TCR sequencing (scTCR‐seq) analysis (Figure S4H). *Pdcd5* ^−/−^ CD8^+^ T cells showed increased TCR diversity and different abundance of TRAV and TRBV usage compared with WT cells (Figure S4H and data not shown). It suggests that *Pdcd5* ^−/−^ T cells have defects in antigen‐induced cell expansion. We identified two common clones between WT and *Pdcd5* ^−/−^ T cells (Figure 6C; Figure S4H). In WT cells, both clones were highly expanded and primarily located in the Teff cluster (C0) (Figure 6C). In contrast, in *Pdcd5* ^−/−^ cells, these clones had only one and two cells each and were distributed across the Teff (C0) and Tex (C1) clusters (Figure 6C). The most expanded TCR clone in the *Pdcd5* ^−/−^ group had the same TCRβ usage as the most expanded clone in the WT group (TRBV20‐TRBJ1‐4) (Figure 6C). However, we found four different amino acids in the center of the complementarity‐determining region (CDR)3 of the β chain: polar amino acids (QDNS) in WT T cells and three out of four nonpolar amino acids (PGTG) in *Pdcd5* ^−/−^ T cells (Figure S4I). Notably, both CDR3β and CDR3α (CAAQGTGSKLSF) of the TCR in *Pdcd5* ^−/−^ cells contained two glycines, suggesting more flexible CDR3 loops that may confer cross‐reactivity and the ability to recognize different antigens [35]. As shown in Figure S4I, these two most expanded TCR clones exhibited different cell cluster distributions. *Pdcd5* ^−/−^ cells were predominantly found in the exhausted T cell clusters (C1 and C3), whereas WT cells were primarily located in the Teff cluster (C0). We further compared the transcriptomes of these two expanded clones and found that the *Pdcd5* ^−/−^ clone had a significantly higher score of exhaustion and higher expression of *Tox* and other inhibitory molecules (Figure 6D; Figure S4I). The expression of *Tbx21*, *Zeb2*, *Hopx*, and *Cx3cr1*, however, was significantly lower compared with the WT clone (Figure 6D; Figure S4I). Collectively, both bulk and scRNA‐seq analyses suggest that T cells lacking *Pdcd5* exhibit defective effector cell differentiation and are biased toward an exhausted phenotype.

### 2.5 Pdcd5‐Deficient CD8^+^ T Cells Have Altered Chromatin Accessibility in Effector Signature Genes

The enrichment of “Reactome_HDMs_demethylate_histones” among the down‐regulated genes by GSEA analysis (Figure 6B; Figure S4B) suggests altered epigenetic regulation in *Pdcd5* ^−/−^ T cells. To investigate this further, we performed ATAC‐seq on CD8^+^ Teff cells at 10 dpi, focusing on chromatin accessibility, particularly at the loci of *Tbx21* and other effector signature genes. Compared with WT Teff cells, *Pdcd5* ^−/−^ cells exhibited more regions/genes (386) with decreased chromatin accessibility peaks (386) than increased peaks (112) (Figure 6E). Among the genes with unique accessibility peaks in WT but not cKO CD8^+^ T cells, 61 were concordant with reduced transcription in *Pdcd5* ^−/−^ cells. These included *Hopx*, *Tbx21*, and T‐bet regulated genes such as *Ifng*, *Gzmb*, *Cx3cr1, Klrg1, Lpin1*, and *S1pr5* (Figure 6F; Figure S5A). Consistent with these findings, the decreased accessibility regions in *Pdcd5* ^−/−^ T cells were enriched for GSE9650_effector_vs_memory_CD8_Tcell_up, as shown by GSEA analysis (Figure 6G). These data suggest that PDCD5 is essential for facilitating chromatin architecture readiness in CD8^+^ Teff cells.

### 2.6 PDCD5 Interacts with PRDM9 and Promotes Its Activity in H3K4me3 Modification During CD8^+^ Teff Cell Differentiation

We previously found that PDCD5 can prevent TOX2 degradation and promote its indirect role in regulating H3K4me3 modification at the *Tbx21* loci in thymic iNKT cells [14]. However, in *Pdcd5* ^−/−^ conventional CD8^+^ T cells, we did not observe a similar decrease in TOX2 levels (Figure S5B), suggesting that the regulation of TOX2 by PDCD5 is cell type‐dependent. Additionally, PDCD5 has been shown to facilitate the degradation of histone deacetylase HDAC3 in tumor cells and endothelial cells [10, 11]. Since HDAC3 suppresses gene programs associated with CD8^+^ Teff cell differentiation early during activation [6], we examined whether HDAC3 levels in T cells are altered in the absence of PDCD5. As shown in Figure S5C, total and phosphorylated HDAC3 levels, as well as the cytoplasmic/nuclear distribution of HDAC3, were comparable between WT and *Pdcd5* ^−/−^ CD8^+^ T cells at 10 dpi. Furthermore, the acetylation level of H3K27 (H3K27ac) was also unchanged in *Pdcd5* ^−/−^ T cells (Figure S5D).

**FIGURE 7:**
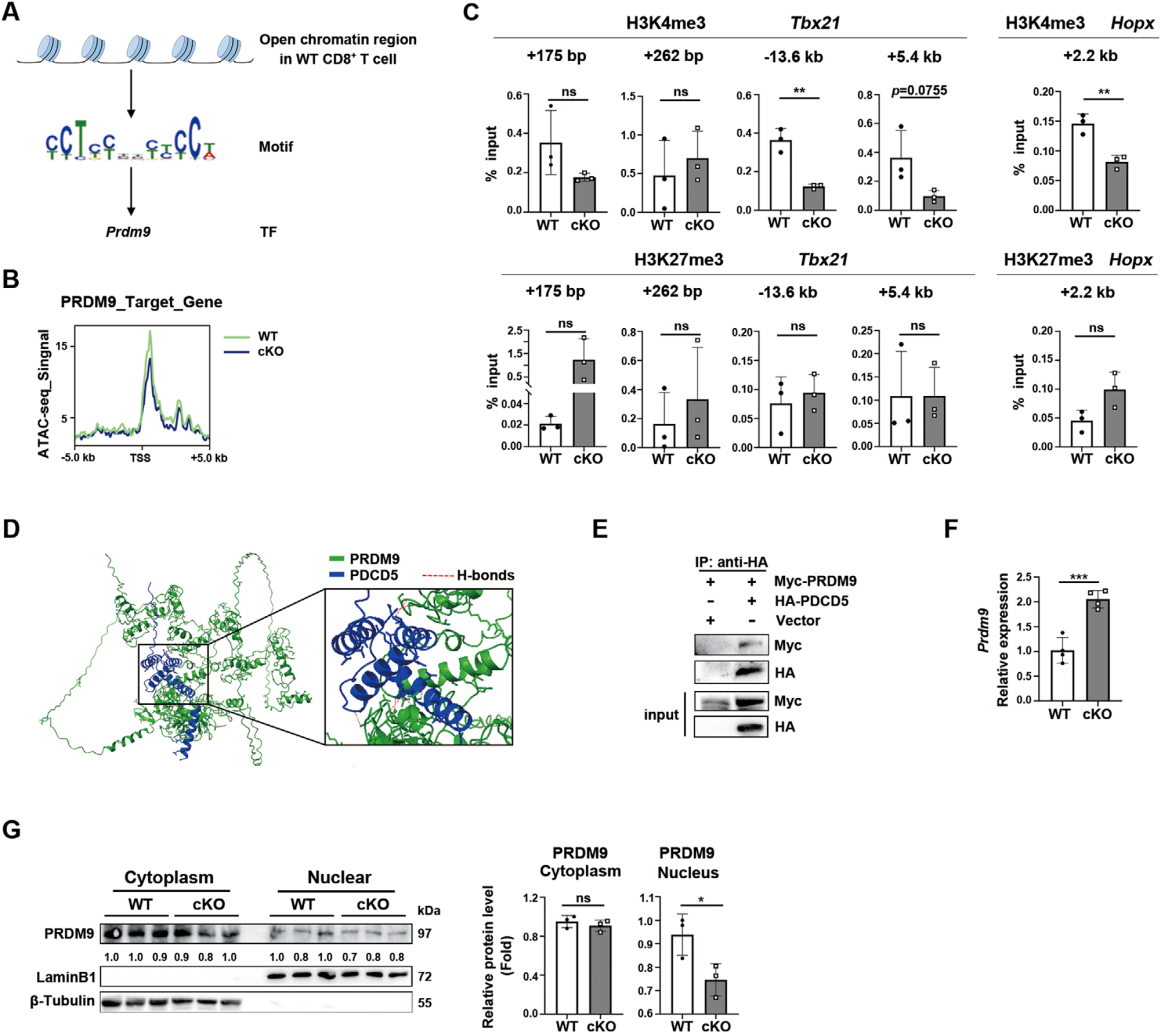
PDCD5/PRDM9 axis in facilitating H3K4me3 modification of *Tbx21* and *Hopx*. (A) Identification of PRDM9 binding motif in unique WT cells‐derived ATAC‐seq data by MEME Suite. (B) Genome‐wide comparison of PRDM9 binding in WT and Pdcd5^−/−^ CD8^+^ T cells. (C) ChIP‐qPCR of the H3K4me3 and H3K27me3 modification in the promoter regions of *Tbx21* and *Hopx* in CD8^+^ T cells purified from the spleen of WT and cKO mice at 10 days after LCMV Cl‐13 infection. Data are representative of 2 independent experiments. (D) AlphaFold‐predicted interaction of a PRDM9 (green)‐PDCD5 (dark blue) complex. The best‐predicted model is shown. The close interaction part is magnified on the right. The red dotted line represents the H‐bond between amino acid residues. (E) 293T cells were co‐transfected with Flag‐HA‐PDCD5 and plasmids. The interaction of PDCD5 and PRDM9 was measured by immunoprecipitation and western blotting. Data are representative of 2 independent experiments. (F) Quantitative RT‐PCR analysis of *Prdm9* mRNA in WT and *Pdcd5* ^−/−^ CD8^+^ T cells at 10 dpi. (G) Western blotting analysis of cytoplasm and nuclear PRDM9 in WT and *Pdcd5* ^−/−^ CD8^+^ T cells. Gray values of the indicated proteins were determined for statistical analysis on the right. Data are representative of two independent experiments. The student's *t*‐test was used for statistical analysis. Mean ± SD, *p < 0.05, **p < 0.01, ***p < 0.001, ns, not significant.

We then utilized the MEME Suite [36] to analyze ATAC‐seq data and determine whether other transcription factors are associated with differential chromatin accessibility. We found an enrichment of PRDM9 binding motifs (*p* = 3.71×10^−4^) in WT relative to KO T cells (Figure 7A,B). PRDM9 is a histone methyltransferase critical for defining DNA double‐strand breaks during meiosis [37, 38]. Knocking down *Prdm9* in CD8^+^ T cells using shRNA led to an increased frequency of KLRG1^−^CD127^+^/CD62L^+^CD127^+^ cells and a decreased frequency of CD62L^−^CD127^−^ cells 7–21 days after LCMV Armstrong infection [39]. However, the mechanism by which PRDM9 regulates Teff cell differentiation remains unclear. PRDM9 is known to generate trimethylation on lysine 4 (H3K4me3) and lysine 36 (H3K36me3) of histone H3 [37, 38]. Since we identified PRDM9 binding motifs in the promoter regions of *Tbx21* and *Hopx* genes (Figure S5E), we performed ChIP‐qPCR analysis. Compared with WT cells, *Pdcd5* ^−/−^ CD8^+^ Teff cells had significantly reduced H3K4me3 modification in the promoter regions of *Tbx21* and *Hopx* (Figure 7C). In contrast, H3K27me3 deposition within these gene loci was comparable between the two groups (Figure 7C). We further explored whether PRDM9 could bind to these genes and direct histone modification. Compared with WT cells, *Pdcd5* ^−/‐^ CD8^+^ T cells showed a slightly reduced level of PRDM9 binding at the *Hopx* locus but not at the *Tbx21* locus (Figure S5F and data not shown). Collectively, these results suggest that the lysine methyltransferase activity of PRDM9 is diminished in *Pdcd5* ^−/−^ CD8^+^ T cells.

We next examined whether PDCD5 interacts with PRDM9 and affects its mRNA/protein levels and subcellular distribution of PRDM9. Both AlphaFold prediction and co‐immunoprecipitation experiments revealed an interaction between PDCD5 and PRDM9 (Figure 7D,E). Specifically, AlphaFold predicted eleven intermolecular hydrogen bonds, including Cys301 (PRDM9)‐to‐Arg79 (PDCD5), Arg40 (PDCD5)‐to‐Tyr304 (PRDM9), Ser42/Gln46 (PDCD5)‐to‐Gln363 (PRDM9), and Lys68 (PDCD5)‐to‐Glu285 (PRDM9). These interactions are located in the PRDM9 PR/SET domain, which mediates the trimethylation of surrounding nucleosomes on lysine of H3 [40] (Figure 7D). Compared with WT cells, *Pdcd5* ^−/−^ CD8^+^ T cells exhibited elevated *Prdm9* mRNA but reduced nuclear PRDM9 protein (Figure 7F,G). The cytoplasmic levels of PRDM9 were comparable between the two groups (Figure 7G). Collectively, these data indicate that PDCD5 interacts with PRDM9 and promotes its nuclear distribution and activity at critical effector gene loci in CD8^+^ T cells.

## 3 Discussion

Epigenetic regulation at the DNA and histone levels is crucial for determining the fate of activated CD8^+^ T cells [41, 42, 43]. In the present study using the LCMV chronic infection mouse model, we found that PDCD5 is upregulated and translocated to the nucleus in activated and proliferating T cells. These changes in PDCD5 are required for the proper differentiation, expansion, and function of antigen‐specific CD8^+^ Teff cells at 10 days post‐LCMV infection. Loss of PDCD5 reduced chromatin accessibility and subsequent expression of the master transcription factor *Tbx21* and its regulated genes. This led to decreased multicytokine production and granzyme B expression, ultimately impairing antiviral responsiveness. The impact of PDCD5 on memory/exhausted CD8^+^ T cell formation at 10 dpi is less clear. Analysis of antigen‐specific gp33 Tet^+^ T cells revealed a decreased frequency of KLRG1^lo^CD127^hi^ cells in cKO mice. The scTCR‐seq further showed that common T cell clones highly expanded in WT cells after infection were rarely found in the *Pdcd5* ^−/−^ group. In contrast, the few expanded T cell clones (with higher numbers of glycine in their CDR3 loops) in the *Pdcd5* ^−/−^ group were unique and predominantly distributed in Tex and Tex_cycling clusters. Upregulated transcription of multiple inhibitory molecules and expansion of Tex cell clusters were also observed in the bulk RNA‐seq analysis of *Pdcd5* ^−/−^ T cells. Interestingly, we did not detect significantly increased cell proliferation or PD‐1 protein upregulation by flow cytometry in these cells, suggesting that additional mechanisms directly or indirectly influenced by PDCD5 may affect the viability and/or migration of exhausted T cells in *Pdcd5* cKO mice.

Graded expression of T‐bet facilitates T cell proliferation and directs the transcriptional program required for effector cell differentiation and function, with the help of other transcription factors such as Zeb2, which is induced by T‐bet [23, 42, 44, 45, 46, 47, 48, 49, 50]. Increasing H3K4me3 marks and decreasing H3k27me3 marks have been reported at the promoter region of *Tbx21* during the process of naïve T‐cell activation and differentiation [43, 51, 52]. BATF and Ezh2 are essential for promoting the loss of H3K27me3 at *Tbx21* loci [52, 53]. However, the molecules that promote H3K4me3 modification of *Tbx21* are less clear. We found that *Pdcd5*‐deficient CD8^+^ T cells had reduced H3K4me3 modification at the enhancer and promoter regions of *Tbx21*. The H3K27me3 modification was not affected by the loss of PDCD5. We further showed that PDCD5 interacts with PRDM9 and promotes its H3K4 methyltransferase activity at *Tbx21* loci.

In addition to *Tbx21*, *Pdcd5* ^−/−^ CD8^+^ T cells at 10 days post‐LCMV Cl‐13 infection also exhibited reduced transcription of *Hopx* (Homeodomain only protein). Hopx is a transcription co‐factor highly expressed in effector CD8^+^, CD4^+^ T cells, and peripheral regulatory T cells [54, 55, 56, 57]. Early induction of Hopx identifies an effector precursor subset in CD4^+^ T cells with imprinted epigenetic instructions for subsequent terminal differentiation [27]. It is reasonable to speculate that Hopx^hi^ cells also mark a “pre‐effector” stage in CD8^+^ T cells. EZH2 may be involved in the negative regulation of Hopx in mesenchymal stem cells [54]. However, the regulation of Hopx expression in T cells remains unclear. We found that the promoter region of *Hopx* contains a PRDM9 binding motif. *Pdcd5* ^−/−^ CD8^+^ T cells exhibited reduced H3K4me3 modification and transcription of *Hopx*. Together with the changes at *Tbx21* loci, these data indicate that PDCD5 facilitates the formation of the epigenetic landscape via PRDM9 at the early stage of Teff differentiation.

PRDM9 is a member of the PR domain‐containing family of lysine methyltransferase, featuring an array of C2H2 zinc fingers for sequence‐specific DNA binding [58, 59]. PRDM9 overexpression in HEK293T cells increases global levels of both H3K4me3 and H3K36me3 modification [60]. The methyltransferase activity of PRDM9 is essential for double‐strand break formation at meiotic recombination hotspots [58]. PRDM9 is also upregulated in various cancer types and can mediate lysine methylation in histone and nonhistone proteins [61, 62]. In T cells, PRDM9 is expressed in CD8^+^ but not CD4^+^ cells (https://www.genecards.org/cgi‐bin/carddisp.pl?gene = PRDM9). However, the roles of PRDM9 in CD8^+^ T cells remain unclear. Recently, it was reported that the knockdown of *Prdm9* in CD8^+^ T cells by shRNA led to an increased frequency of KLRG1^−^CD127^+^/CD62L^+^CD127^+^ memory‐like CD8^+^ cells and decreased frequency of CD62L^−^CD127^−^ effector‐like cells at 7–21 days post‐LCMV Armstrong infection [39]. Our findings using the LCMV Cl‐13 infection model suggest that PRDM9 promotes H3K4me3 modification at the loci of critical transcription factors such as *Hopx* and *Tbx21*, thus facilitating Teff cell differentiation.

PDCD5 has been found to be phosphorylated at Ser119 and quickly translocated into the nucleus in tumor cells in response to genotoxic stress [10, 63]. Nuclear PDCD5 then interacts with p53, preventing its degradation and nuclear export [64]. We also observed increased nuclear distribution of PDCD5 in CD8^+^ T cells upon stimulation with CD3‐ and CD28‐specific antibodies, likely via activation‐induced phosphorylation of PDCD5. As *Pdcd5*‐deficient T cells exhibited reduced nuclear but not cytoplasmic fractions of PRDM9, and PDCD5 can interact with PRDM9, these results indicate that PDCD5 promotes the nuclear distribution of PRDM9, thereby facilitating its methyltransferase activity at histone proteins. Whether PDCD5 prevents the nuclear export of PRDM9 in a manner similar to that of p53 remains to be investigated.

Taken together, our findings reveal a pivotal role of the PDCD5/PRDM9 axis in epigenetic reprogramming at the early stage of effector CD8^+^ T cell fate determination in the LCMV chronic infection model. Whether the overexpression of PDCD5 can enhance effector T cell differentiation in the context of antitumor immunotherapies remains to be tested. In addition, upregulated PDCD5 expression has been observed in peripheral mononuclear cells from patients with multiple sclerosis and Hashimoto's Thyroiditis [65]. Thus, further understanding the roles in effector T cell differentiation in these autoimmune disorders is of significant importance.

## 4 Materials and Methods

### 4.1 Mice

To specifically knock out *Pdcd5* in T cells, we bred *Pdcd5* ^fl/fl^ mice with *Cd4*‐Cre transgenic mice (*Pdcd5* ^fl/fl^ *Cd4*‐Cre, cKO). *Pdcd5* ^fl/fl^ mice and *Cd4*‐Cre transgenic mice were kindly provided by Professors Yingyu Chen (Peking University Health Science Center, China) and Lilin Ye (Army Medical University, China), respectively. *Rag1* ^−/−^ mice were kindly provided by Prof. Hong Tang (University of Chinese Academy of Sciences). C57BL/6 congenic mice (CD45.1^+^) were purchased from Peking University Health Science Center (Beijing, China). All the mice were on the C57BL/6 background and were used at 8–12 weeks of age. The animals were kept in a specific pathogen‐free facility at Peking University (Beijing, China). The experimental procedures on the use and care of animals had been approved by the Ethics Committee of Peking University (LA2022106).

### 4.2 LCMV Infection Model and Viral Titer Quantification

Mice were infected with LCMV clone 13 strain (LCMV Cl‐13) (4 × 10^5^ plaque‐forming units (PFU), intraperitoneally (i.p.) or 2.5×10^5^ PFU, intravenously (i.v.)) for chronic infections. For the in vivo CD4^+^ T cell clearance model, the mice were intraperitoneally injected with 200 µg anti‐mouse CD4 (GK1.5, Bio X Cell, Lebanon, NH, USA) on days −1 and +1.

The LCMV viral loads in blood samples were quantified by qPCR as previously described [66]. Briefly, the stock LCMV was titrated with the Vero cell plaque assay. Total RNA from the stock virus and blood was extracted by TIANamp Virus RNA Kit (Tiangen, Beijing, China). The amount of LCMV glycoprotein (GP) mRNA present in the blood was measured by qPCR, and a series of dilutions of stock LCMV mRNA was used as a standard curve.

## Author Contributions

Qing Ge, Dan Lu, Lixue Jin, Xin Zhang, and Jingyi Wang designed the research, performed the research, analyzed data, and wrote the paper. Lixue Jin, Xin Zhang, and Jingyi Wang contributed equally. Yujia Wang, Ke Wang, and Zhuolin Wang performed the research and helped with flow cytometry. Pingzhang Wang, Xiuyuan Sun, and Jie Hao contributed animals, critical reagents, and technical support. Rong Jin provided critical suggestions. All the authors reviewed the manuscript.

## Conflicts of Interest

The authors declare no conflicts of interest.

### Peer Review

The peer review history for this article is available at https://publons.com/publon/10.1002/eji.202451388.

## Supporting information



Supplementary Material

## Data Availability

All sequencing data generated in this study have been deposited at NCBI's Gene Expression Omnibus (GEO) repository with the RNA‐seq data being accessible through GEO Series accession number GSE231510. The public scRNA‐seq data were obtained from GSE164522, GSE156728, and GSE155223. The public bulk RNA‐seq data were obtained from GSE119943. Other materials and methods are listed and described in supplemental materials.
