# Supplementary material for: Epigenetic Regulation of CD8+ Effector T Cell Differentiation by PDCD5
Source: Eur J Immunol. 2025 Mar 20;55(3):e202451388. doi: 10.1002/eji.202451388 (PMC11924876; doi:10.1002/eji.202451388)
Supplement: Supplementary file 1 — Supplementary Material [file EJI-55-e202451388-s001.docx]

Title: Epigenetic regulation of CD8^+^ effector T cell differentiation by PDCD5

Running title: PDCD5 regulates effector T differentiation

Lixue Jin^1,4^, Xin Zhang^1,4^, Jingyi Wang^1,4^, Yujia Wang^1^, Ke Wang^1^, Zhuolin Wang^1^, Pingzhang Wang^1^, Xiuyuan Sun^1^, Jie Hao^1^, Rong Jin^1^, Dan Lu^2^*, Qing Ge^1,3^*

**Supplemental Figure legends**

Figure S1 Mice carrying a T cell-specific deletion of *Pdcd5* had reduced CD44^hi^ peripheral CD8^+^ T cells under steady-state conditions and reduced effector CD8^+^ T cells 10 days after LCMV Cl-13 infection. (A-C) *Pdcd5* expression in various T cell subsets obtained from colorectal cancer patients- (A) (CD8^+^ and CD4^+^ T cells from GSE164522, analyzed and plotted using cancer-pku.cn:3838/CRLM), esophageal and ovarian cancer patients (B) (CD8^+^ T cells from GSE156728), a COVID-19 patient, and a healthy donor (C) (CD8^+^ T cells from GSE155223) derived scRNA-seq data. (D-E) Quantitative RT-PCR (D) and western blotting (E) analysis of PDCD5 expression in purified CD4^+^ and CD8^+^ T cells in the lymph nodes and spleen of *Pdcd5*^fl/fl^ (WT) and *Pdcd5*^fl/fl^*Cd4*-Cre (cKO) mice. β-actin was used as the housekeeping gene control in quantitative RT-PCR. (F) Flow cytometry comparison of CD4^-^CD8^-^ (DN), CD4^+^CD8^+^ (DP), CD4^+^CD8^-^ (CD4 SP), CD4^-^CD8^+^ (CD8 SP) thymocytes in WT and cKO mice. (G) Flow cytometry analysis of the percentages and numbers of total (left panel) and CD44^hi^ (right panel) CD4^+^ and CD8^+^ T cells in the lymph nodes of WT and cKO mice. Data are representative of 2-3 independent experiments. Student’s *t*-test was used for statistical analysis. Mean ± SD, * *P* < 0.05, ** *P* < 0.01, *** *P* < 0.001, **** *P* < 0.0001, ns, not significant.


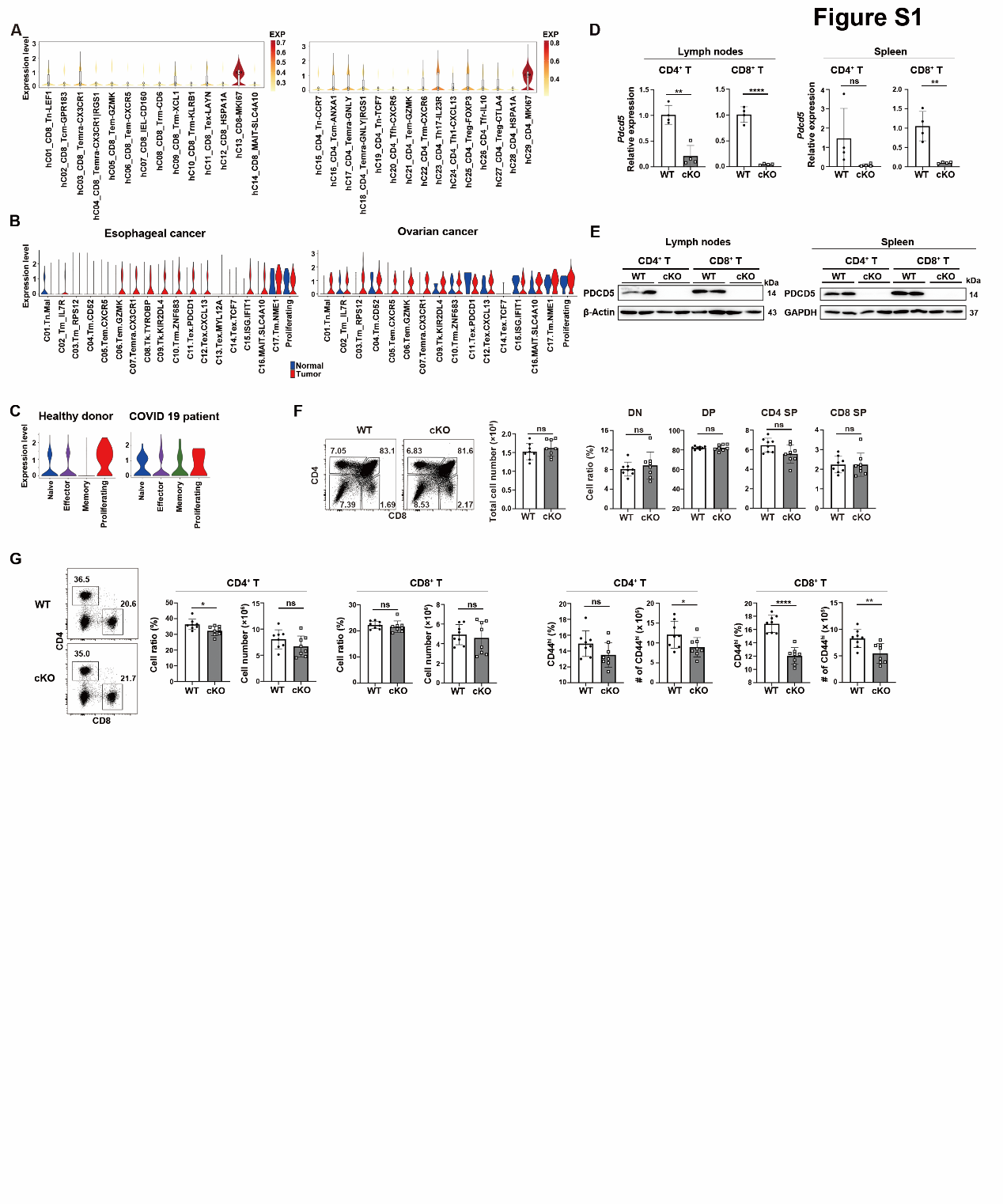


Figure S2 *Pdcd5*^-/-^ CD8^+^ T cells had reduced antiviral responses at 30 days post LCMV Cl-13 infection. WT and cKO mice were infected with LCMV Cl-13 at 4×10^5^ plaque-forming units (PFU) via intraperitoneal (i.p.) injection (A-C) and 2.5x10^5^ PFU via intravenous (i.v.) injection (D-F). (A) Quantitative RT-PCR of LCMV-glycoprotein (GP) mRNA in various tissues. WT group had 2-4 mice and cKO group had 2-6 mice for the analysis. (B) Flow cytometry analysis of the percentages and cell numbers of CD44^hi^CD62L^lo^, CD44^hi^CD62L^hi^, CD44^lo^CD62L^hi^ cells in CD8^+^ T cells in the spleen and liver of WT and cKO mice. (C) Flow cytometry analysis of PD-1 and TIM-3 expression in splenic and liver CD8^+^CD44^hi^ T cells. (B-C) had 4 and 6 mice for WT and cKO group, respectively. (D) Quantitative RT-PCR of LCMV-glycoprotein (GP) mRNA in various tissues. Five mice in each group. (E) Comparison of the percentage and cell number of Tet^+^CD8^+^ T cells in the spleen and liver of WT and cKO mice. (F) Flow cytometry analysis of PD-1, TIM-3 expression in splenic and liver CD8^+^CD44^hi^ T cells. (E-F) had 7 mice in each group. Data are representative of 2 independent experiments. Student’s *t*-test was used for statistical analysis. Mean ± SD, * *P* < 0.05, ** *P* < 0.01, *** *P* < 0.001, **** *P* < 0.0001, ns, not significant.


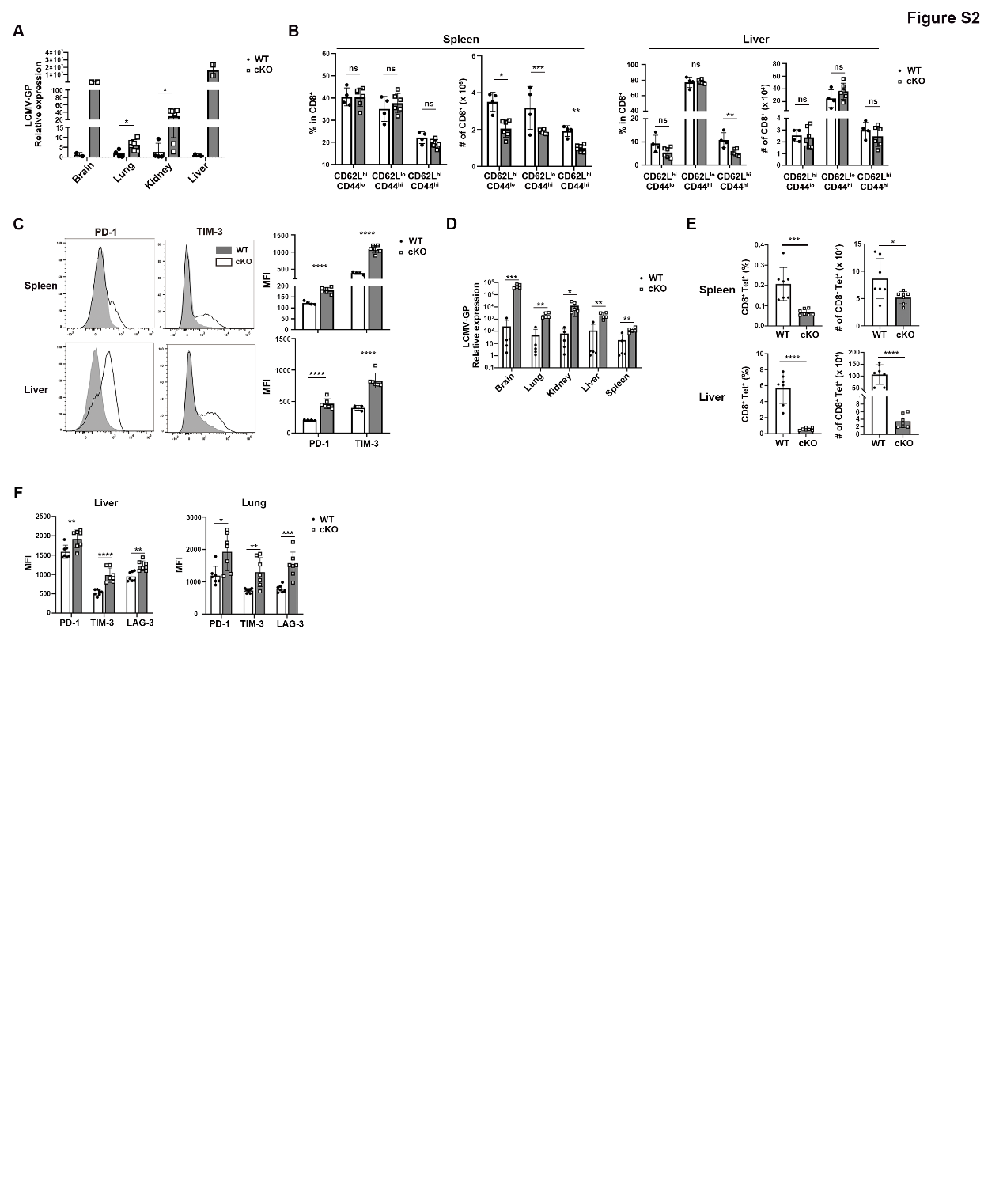


Figure S3 PDCD5 regulated CD8^+^ Teff cell differentiation in a cell-intrinsic manner. (A-D) CD4-specific antibody was intraperitoneally injected into WT and cKO mice 1 day prior and 1 day after LCMV Cl-13 infection. The diagram is shown in (A). The body weight changes between WT and cKO mice post-infection are compared in (B). The spleen and liver were then harvested at 10 dpi for flow cytometry analysis. A comparison of the percentages and cell numbers of total CD8^+^ T cells between WT and cKO mice is shown in (C) with the efficiency of CD4 depletion plotted in the left panel with the red rectangles. Flow cytometry analysis of the percentages of indicated CD8^+^ T cell subsets is shown in (D). (E-G) Mixed BM chimeric mice were generated by coinjecting cKO CD45.2^+^ and WT CD45.1^+^ BM cells (1:1 ratio) into lethally irradiated CD45.1^+^ WT mice. The chimeric mice were infected with LCMV Cl-13 at 7 weeks after reconstitution and were harvested 10 days later. The percentages of splenic and liver Tet^+^ CD8^+^ T cells, total CD8^+^ T cells (E), and indicated CD8^+^ T cell subsets (F) were calculated and compared between WT (CD45.1^+^)- and cKO (CD45.2^+^)-derived cells. The frequencies of Ki67^+^ cells compared between WT and cKO CD8^+^ T cells in the spleen are shown in (G). Data are representative of 2 independent experiments. Student’s *t*-test was used for statistical analysis. Mean ± SD, * *P* < 0.05, ** *P* < 0.01, *** *P* < 0.001, **** *P* < 0.0001.


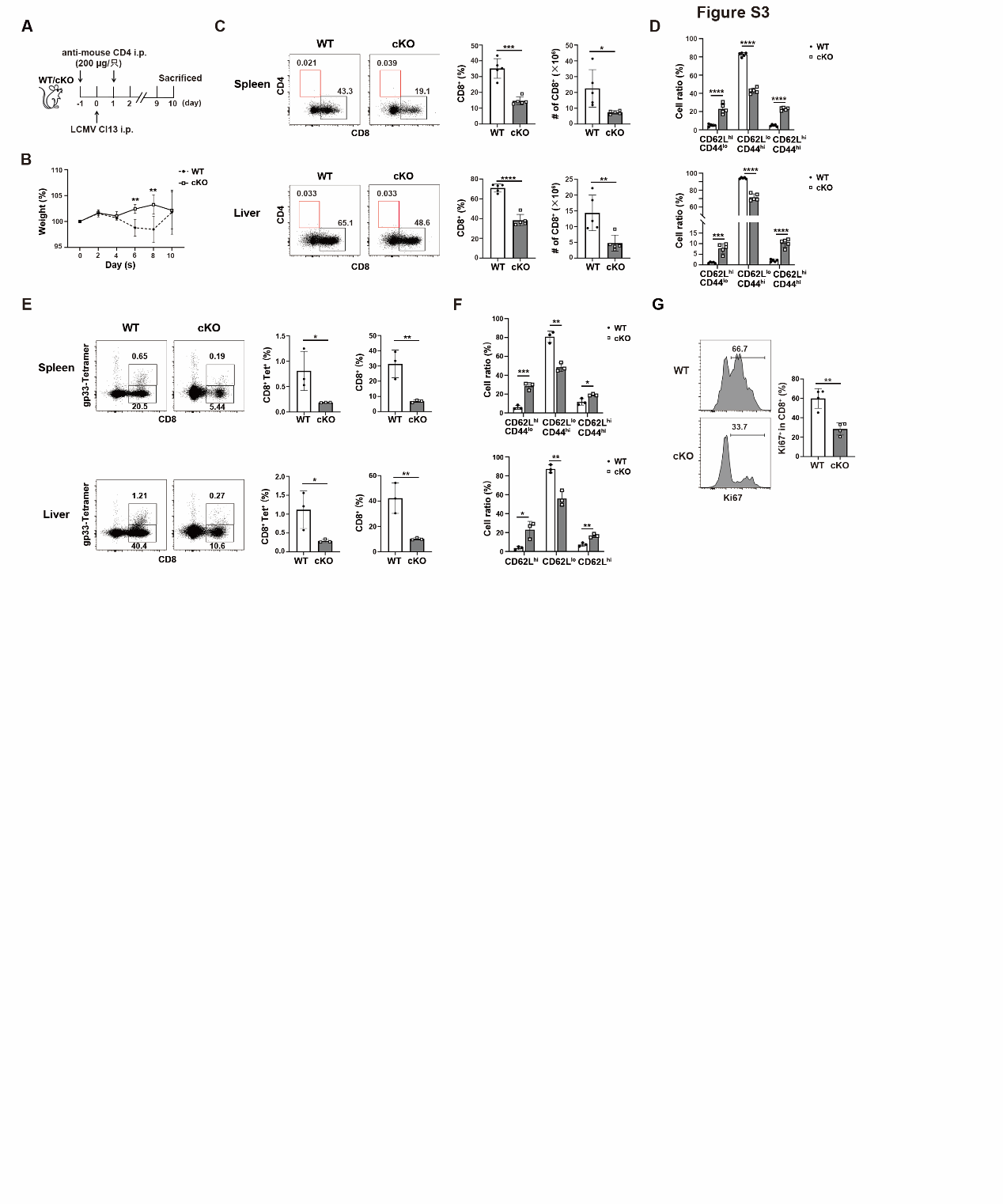


Figure S4 Impaired effector cell differentiation in the transcriptome of *Pdcd5-*deficient T cells. WT and *Pdcd5* cKO mice (2 mice in each group) were infected with LCMV Cl-13 at 4×10^5^ plaque-forming units (PFU) via intraperitoneal (i.p.) injection. Ten days later, splenic CD8^+^ T cells were purified by anti-CD8 antibody-labeled magnetic beads. The cells were then subjected to bulk RNA-seq (A-B), scRNA-seq, and scTCR-seq (C-I). (A) Heatmap showing the expression of T cell differentiation-related genes (left panel) and T-bet-induced genes (right panel) obtained from the bulk RNA-seq. (B) Heatmap showing the expression of leading-edge genes (bulk RNA-seq) listed in reactome_HDMS_demethylate_histones together with chromatin-remodeling protein genes *Chd3* and *Chd9*. (C) UMAP visualization of CD8^+^ T cell clusters obtained from scRNA-seq analysis. (D) Violin plots showing cell cycle gene expression changes (reported as normalized G2M and S phase scores). (E) Heatmap showing the row-scaled expression of selected signature genes for each cluster. Teff, effector T cells, Tex, exhausted T cells, Tactiv, activated T cells, Tem, effector memory T cells, Tpro, resting or pre-exhausted progenitor cells. (F) Violin plot showing *Pdcd5* expression in each cluster. (G) Comparison of T cell subset frequencies. The y-axis shows Log2 of the ratio of the frequencies of the indicated clusters. The dot size represents the frequency of each cluster within its sample. The x-axis and dot color show indicated clusters. (H) Comparison of TCR clonotypes. Left, UMAP plots of T cell samples with cells colored according to the size of their TCR clonotypes. Middle, comparison of TCR diversity between WT and *Pdcd5*^-/-^ T cells. Right, comparison of top 20 TRAV usage between WT and *Pdcd5*^-/-^ T cells. (I) Comparison of the most expanded TCR clones between WT and *Pdcd5*^-/-^ T cells. Left, Dot plot comparing the distribution (proportion) of the most expanded TCR clones in each cluster. The dot size represents the frequency of each cluster within the most expanded TCR clones. WT: TRBV20-TRBJ1-4-TRBC1 (CDR3β: CGARDQDNSNERLFF)/TRAV5N-4-TRAJ21-TRAC (CDR3α: CAASPNYNVLYF); cKO: TRBV20-TRBD1-TRBJ1-4-TRBC1 (CDR3β: CGARDPGTGNERLFF)/TRAV4-3-TRAJ58-TRAC (CDR3α: CAAQGTGSKLSF). Right, Violin plot comparing the exhaustion scores of the most expanded TCR clones between WT and cKO CD8^+^ T cells. Student’s *t*-test was used for statistical analysis. **** *P* < 0.0001.


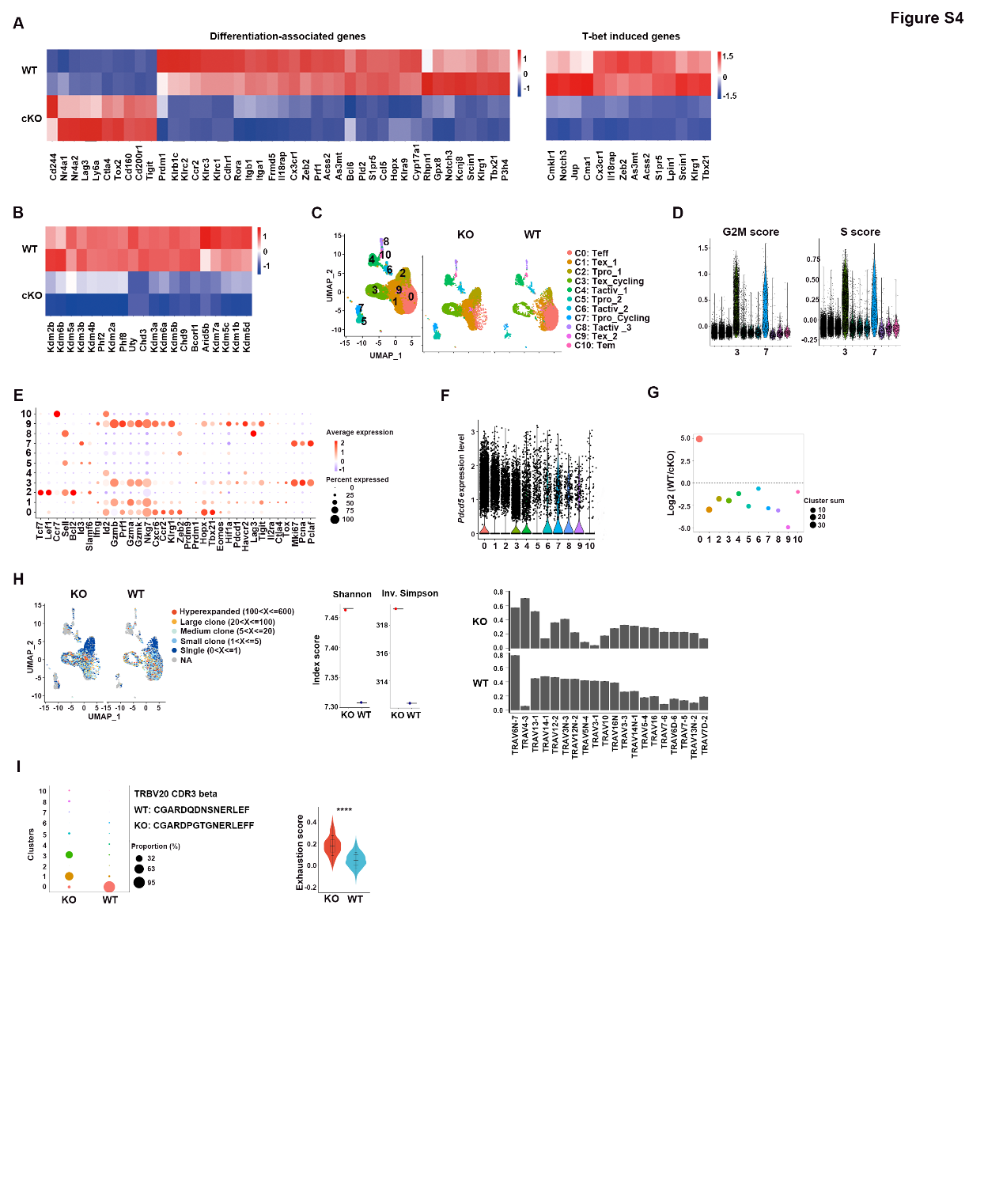


Figure S5 Reduced chromatin accessibility of effector signature genes in *Pdcd5*^-/-^ CD8^+^ T cells 10 days after LCMV Cl-13 infection. WT and cKO mice were infected with LCMV Cl-13 at 4×10^5^ PFU via intraperitoneal injection. Ten days later, the splenic CD8^+^ T cells were purified by magnetic bead-based sorting and were subjected to ATAC-sequencing (3 mice per group). (A) Integrative Genomics Viewer (IGV) analysis of ATAC-seq coverage of the indicated genes. (B) Western blotting analysis of TOX2 in CD8^+^ T cells at 10 dpi. Gray values of the indicated proteins were determined for statistical analysis at the bottom. (C) Western blotting analysis of total, phosphorylated, cytoplasmic, and nuclear HDAC3 in CD8^+^ T cells at 10 dpi. Gray values of the indicated proteins were determined for statistical analysis at the bottom. (D) Western blotting analysis of acetylated H3K27 (H3K27ac) in CD8^+^ T cells at 10 dpi. Gray values of the indicated proteins were determined for statistical analysis at the bottom. The experiments were repeated for 2-3 times and representative data are shown (C-D). Student’s *t*-test was used for statistical analysis. Mean ± SD, ** *P* < 0.01, ns, not significant. (E) Predicted PRDM9 binding sites at *Tbx21* and *Hopx* gene loci. (F) ChIP-qPCR of PRDM9 in the predicted binding site of *Hopx* in CD8^+^ T cells purified from the spleen of WT and cKO mice at 10 days after LCMV Cl-13 infection.


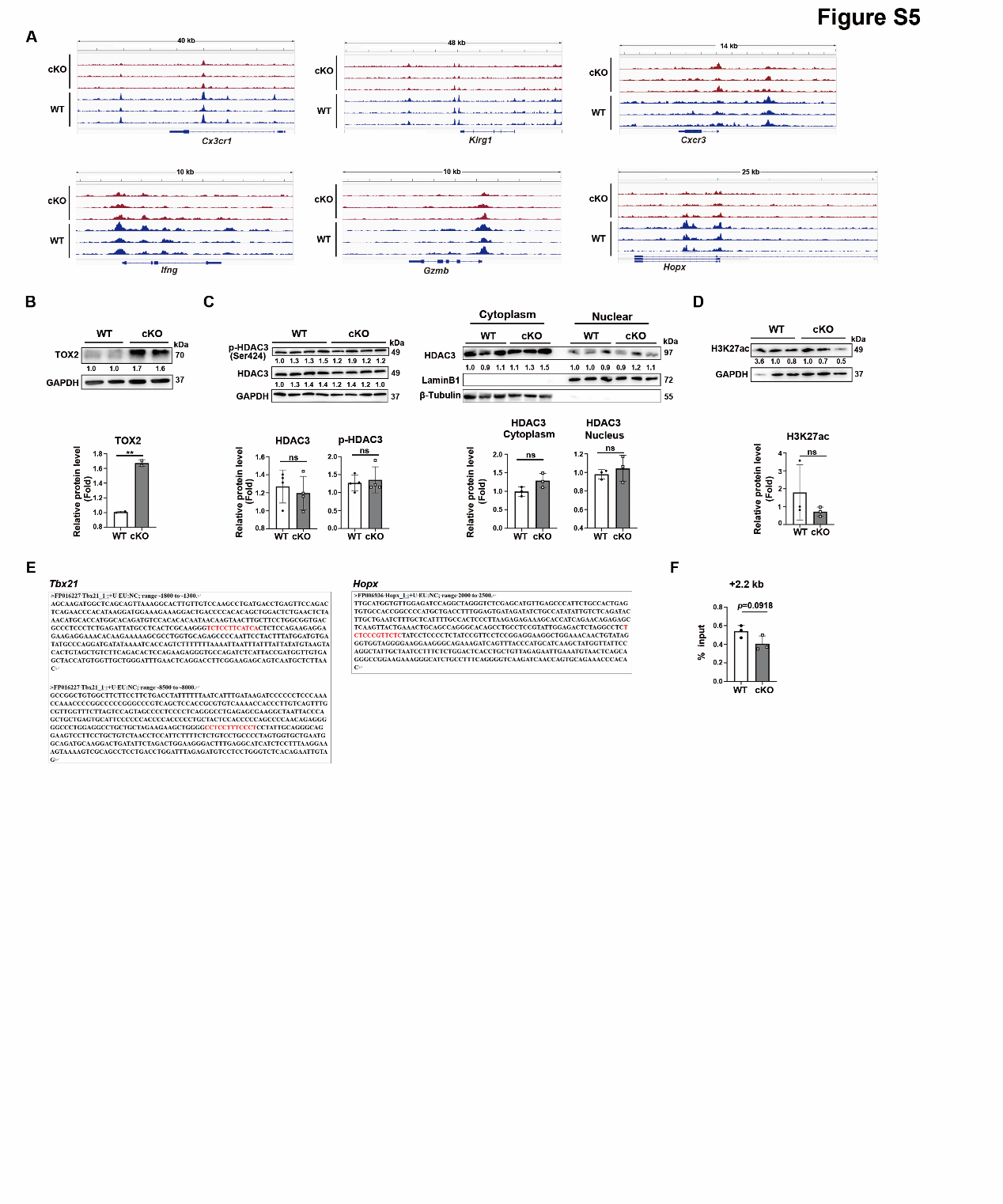


**Supplemental materials and methods**

*Antibodies*

Primary antibodies against Myc tag, GAPDH, AKT, phosphorylated AKT, ERK1/2, phosphorylated ERK1/2, phosphorylated p65, p70S6K, phosphorylated p70S6K, GLUT1, c-Myc, IRF4, BCL6, STAT1, phosphorylated STAT1, STAT3, phosphorylated STAT3, STAT4, and phosphorylated STAT4 were purchased from Cell Signaling Technology ((Danvers, MA, USA), those against hemagglutinin (HA) and β-actin were from Proteintech (Rosemont, IL, USA), the antibodies against PDCD5 and T-bet were from Abcam (Cambridge, MA, UK), and the antibodies against β-Tubulin and LaminB1 were from BioWorld (Irving, Texas, USA).

The following antibodies used for flow cytometry were purchased from CD4 (GK1.5, BD Biosciences, Biolegend), CD8 (53-6.7, BD Biosciences, Biolegend), CD44 (IM7, eBioscience, Biolegend), CD62L (MEL-14, BD Biosciences, Biolegend), CD25 (PC61, BD Biosciences, eBioscience), CD122 (TM-β1, Biolegend), PD-1 (29F.1A12, Biolegend), TIM-3 (B8.2C12, Biolegend), LAG-3 (C9B7W, BD Biosciences), T-bet (04-46, eBioscience), SLAMF6 (13G3-19D, eBioscience), CD127 (A7R34, Biolegend), KLRG1 (2F1, Biolegend), CX3CR1 (SA011F11, Biolegend), Blimp-1 (5E7, BD Biosciences), EOMES (DAN11MAG, eBioscience), Tcf-1 (S33-966, BD Biosciences), IFN-γ (XMG1.2, BD), TNF-α (MP6-XT22, eBioscience, Biolegend), Granzyme B (GB11, Biolegend), CD107a (1D4B, BD, Biosciences Biolegend), Ki67 (SoIA15, eBioscience) and T-Select H-2Db LCMV gp33 Tetramer-KAVYNFATC-PE (MBL International, Woburn, MA, USA).

*Bone marrow chimera*

CD45.1^+^ WT recipients were subjected to lethal irradiation (900 rads). Six hours later, WT (CD45.1^+^) and PDCD5 cKO (CD45.2^+^) bone marrow cells were mixed at a 1:1 ratio, and a total of 5 × 10^6^ cells were injected intravenously into recipient mice. After 7 weeks, the mixed bone marrow chimeric mice were infected with the LCMV Cl-13 strain.

*Adoptive transfer experiment in RAG1^-/-^ mice*

The CD8^+^CD44^lo^CD62L^hi^CD25^-^ T cells from the lymph nodes of WT and *Pdcd5* cKO mice were purified by flow cytometry and labeled with 2 μM CFSE at room temperatur for 5 minutes. The T cells at 1 x 10^6^ were then adoptively transferred into adult syngeneic RAG1^-/-^ mice. Six days later, lymph node cells from RAG1^-/-^ mice were harvested and analyzed by flow cytometry.

*Cell preparation and cell sorting*

To obtain hepatic mononuclear cells, the liver cell suspension was purified with a Percoll gradient (GE Healthcare, USA). To isolate lymphocytes infiltrating in the lung, the minced tissues were digested with 0.5 mg/ml Collagenase IV (Gibco, USA) and 25 μg/ml DNase I (Meilunbio, China) at 37ºC for 30 min. The tissues were then ground and filtered. A 40% (10 ml)/70% (5 ml) Percoll gradient was used to isolate the lymphocytes.

To obtain cells for RNA-seq or ATAC-seq, CD8^+^ splenic T cells from mice at day 10 after LCMV Cl-13 infection were sorted either by flow cytometry (BD Aria Sorp) or by magnetic bead separation using anti-FITC Microbeads and the autoMACS system (Miltenyi Biotec, Auburn, CA, USA) with positive selection for the CD8 surface antigen according to the manufacturer’s instructions.

*Immunoprecipitation and western blotting*

Cells were washed with ice-cold PBS and lysed on ice for 30 min in RIPA buffer (50 mM Tris-HCl, pH 7.5; 135 mM NaCl; 1% NP-40; 0.5% sodium DOC; 1 mM EDTA; 10% glycerol) or IP lysis buffer (20 mM Tris-HCl, pH 7.5, 150mM NaCl, 1mM EDTA, 1% NP-40) with a mixture of protease inhibitors and phosphatase inhibitors (Roche, Switzerland). Cell lysates were cleared by centrifugation and supernatants were immunoprecipitated with appropriate antibodies. The samples were rocked for 4 hours at 4°C and were then mixed with 50 μl of a 50% slurry of Protein A-Sepharose (GE Healthcare, USA) overnight at 4°C. The immunoprecipitates were washed for five times in IP washing buffer (20 mM Tris-HCl, pH 7.5, 500 mM NaCl, 1 mM EDTA, 1% NP-40) and analyzed by western blotting. The protein bands were detected by chemiluminescence using an enhanced ECL chemiluminescence kit (Biodragon, China) and exposed to Tanon-5200 (Tanon, China).

*ChIP–qPCR*

Ten million CD8^+^ T cells were isolated from the spleen of WT and cKO mice at 10 days post LCMV Cl-13 infection and were subjected to crosslinking for 10 min with 1% formaldehyde in medium and quenching with 1x glycine for each ChIP. Pierce Agarose ChIP Kit (26156, Invitrogen) was used for chromatin fragments preparation, immunoprecipitation, and DNA purification according to the manufacturer’s instructions. Immunoprecipitation with anti-H3K4me3 (9751, Cell Signaling Technology) or anti-H3K27me3 (9733, Cell Signaling Technology) was performed and was followed by qPCR with appropriate primers (Table S1).

*Measurement of cytokine production in vitro*

Cells isolated from the spleen and liver of WT and PDCD5 cKO mice were stimulated in a 24-well plate with 100 ng/ml PMA (Biosharp Life Sciences, Hefei, Anhui, China), 1 μg/ml ionomycin (Abcam, Cambridge, MA, UK), or 50 μg/ml LCMV GP_33-41_ peptide (GL Biochem, Shanghai, China), and 1 μg/ml Brefeldin A (BFA, Biosharp Life Sciences) in complete medium (Rosewell Park Memorial Institute 1640, 10% fetal bovine serum). Cells were incubated for 4 h at 37 °C before intracellular staining of cytokines, granzyme B and CD107a.

*RNA isolation and real-time PCR*

RNA was purified from T cell subsets using TRIzol (Invitrogen). The purity of RNA was verified spectrophotometrically at 260/280 nm. The RNA samples (2 μg) were reverse transcribed into cDNAs using the FastQuant reverse transcription kit (Tiangen, Beijing, China) according to the manufacturer’s instructions. Quantitative real-time PCR was performed using FastStart Universal SYBR Green Master mix (Roche, Basel, Switzerland) on a real-time PCR system (PCRmax Eco, Illumina, USA).

The following PCR primers were used: *Pdcd5*, forward, 5′-CCAACAGGAAGCAAAGCAA-3′, reverse, 5′-TTCAGGCTTTACAAGTGCTAAGTTAC-3′; *β-actin*, forward, 5′-TCGTGCGTGACATCAAAGAG-3′, reverse, 5′-GGATTCCATACCCAAGAAGG-3′; *Tbx21*, forward, 5′-AACACACACGTCTTTACTTTCCA-3′, reverse, 5′-CGTATCAACAGATGCGTACATGG-3′; *LCMV-GP*, forward, 5′-TGCCTGACCAAATGGATGATT-3′, reverse, 5′-CTGCTGTGTTCCCGAAACACT-3′; *Zeb2*, forward, 5′-GAGCAGGTAACCGCAAGTTC-3′, reverse, 5′-TGTTTCTCATTCGG-3′; *Hopx*, forward, 5′-CACCACGCTGTGCCTCATCG-3′, reverse, 5′-CAAAACAGCCTGGGTAAGCC-3; *Prdm9*, forward, 5′-ATATGGAATGGAATCATCGC-3′, reverse, 5′-GTGCTG GGAAAGGTTGTT-3′.

*RNA-seq*

On day 10 of LCMV Cl-13 infection, 2x10^6^ CD8^+^ T cells from the spleen of WT and cKO mice were sorted by flow cytometry. RNA integrity and quantity were measured using the Agilent 2100/4200 system. The library was constructed using the standard Illumina library construction process. After library construction, the concentration of the library was measured by the Qubit® fluorometer. The accurate concentration of the cDNA library was again examined using qPCR. The size distribution of the library was detected by agarose gel electrophoresis. After library preparation and pooling of different samples, the samples were subjected to Illumina sequencing. The raw data (raw reads) of the FASTQ format were first processed through in-house Perl scripts. Paired-end clean reads were aligned to the reference genome using Hisat2. Feature count was used to counting the reads numbers mapped to each gene. EdgeR was used for differential expression analysis. The resulting *P*-values were adjusted using Benjamini and Hochberg’s approach for controlling the false discovery rate. GO and KEGG enrichment analysis of differentially expressed gene sets were implemented by the topG （http://www.bioconductor.org/packages/release/bioc/html/topGO.html） and the KOBAS package, respectively.

*Library construction assay for transposase-accessible chromatin*

On day 10 of LCMV Cl-13 infection, 5x10^4^ CD8^+^ T cells from the spleen of WT and cKO mice were sorted by flow cytometry. The library construction used TruePrepTM DNA Library Prep Kit V2 for Illumina (TD501-01, Vazyme) according to the manufacturer’s instructions.

*ATAC-seq data analysis.*

Adapter sequences and low-quality reads were trimmed from both ends of the raw ATAC-seq reads using fastp (https://github.com/OpenGene/fastp#fastp, v0.23.2). Reads shorter than 15 nt after adapter trimming were discarded. Subsequently, the reads were mapped to the mm10 reference sequence using Hisat2 (http://daehwankimlab.github.io/hisat2/, v2.2.1). A step-wise mapping procedure was applied. (1) The reads that were not aligned to the reference sequence were discarded. (2) The reads with low alignment quality were discarded. (3) The reads that aligned to the mitochondrial sequence were discarded. The peaks were identified using macs2 (https://pypi.org/project/MACS2/, v2.2.7.1). The R package ChIPseeker (1.26.2) was used for peak annotation. The R package clusterProfiler (4.0.2) was used for GO and KEGG enrichment analysis. Visualization of peak distribution along genomic regions of interested genes was performed with the Integrative Genomics Viewer (IGV). Motif enrichment analysis was carried out for the peak regions found in exons using MEME (https://meme-suite.org/meme/doc/meme.html), searching for motif lengths from 8 to 12. Transcription factor (TF) enrichment analysis was carried out for the top10 motif using Tomtom (https://meme-suite.org/meme/tools/tomtom). Heatmaps of ATAC-seq signal was plotted using deeptools (https://deeptools.readthedocs.io/en/develop/, v2.5.3).

*AlphaFold analysis*

The interaction between PRDM9 and PDCD5 was analyzed using AlphaFold (<https://golgi.sandbox.google.com/>) to predict the protein-protein interaction. The AlphaFold-generated structural model provided insights into the spatial arrangement of the proteins and identified potential hydrogen bonds involved in their interaction. To visualize the predicted interaction, the structural model was further processed and visualized using ChimeraX software (version 1.7.1), which allowed for a detailed examination of the binding interface and hydrogen bond formation between PRDM9 (green) and PDCD5 (dark blue).

*scRNA-seq library preparation*

CD8^+^ T cells from the spleen of two WT and two *Pdcd5* cKO mice ten days post LCMV Cl-13 (4x10^5^ PFU, i.p.) infection were purified using CD8 Microbeads (Miltenyi Biotec, Auburn, CA, USA) according to the manufacturer’s instruction. Using single cell 5’ Library and Gel Bead Kit (1000006, 10x Genomics, Pleasanton, CA, USA), Single Cell V(D)J Enrichment Kit, Mouse T Cell (1000071), the cell suspension (1,000 cells/μl, cell viability > 90%) was loaded onto the Chromium single cell controller (10x Genomics) to generate single-cell gel beads in the emulsion according to the manufacturer’s protocol. The libraries were sequenced using an Illumina Novaseq 6000 sequencer.

*scRNA-seq data processing*

The quality of the sequenced reads was evaluated by FastQC. The FASTQ sequenced files were then aligned with the Mm10 mouse transcriptome using Cell Ranger (v3.1.0) and the transcript expression of each cell was quantified. The expression matrix of WT and cKO samples was loaded into R (v4.0.2) and was analyzed using Seurat (v4.0.2). The following criteria was adopted to control the quality of the data. (1) Cells that expressed >200 and < 4000 genes. (2) The percentage of mitochondrial gene < 10. After quality control, the NormalizeData function was used to remove the differences in sequencing depth across cells.

*Batch correction*

The ScaleData function was used to eliminate batch effects caused by batch and mitochondrial gene expression. The harmony (v0.1.0) package was then used to reduce the batch effect.

*Clustering and annotation*

The RunPCA method was used for dimensionality reduction, and the top 20 principal components were selected for downstream analysis. The RunUMAP/RunTSNE function was applied to reduce dimensionality. A shared nearest neighbor (SNN) graph is constructed using the FindNeighbors function, and 11 clusters were identified using the FindCluster method. Based on normalized data, differentially expressed genes were identified by FindMarkers or FindAllMarkers function. The CellCycleScoring function was used to define the cycle state of cells. GSEA was performed by using the R package clusterProfiler.

scTCR-seq analysis

The quality of TCR sequenced reads was evaluated by FastQC. The FASTQ sequenced files were alighted with the refdata-cellranger-vdj-GRCm38-alts-ensembl-4.0.0 using the Cell Ranger VDJ function. The filtered_contig_Annotation files were loaded into R and were merged through the combineTCR function. The scRNA-seq data were combined with scTCR-seq data using the combineExpression function of scRepertoire (v1.3.4). The frequency of clonotypes was categorized by 5 levels (Hyperexpanded, 100 < X ≤ 1000), Large, (20 < X ≤ 100), Medium (5 < X ≤ 20), Small (1 < X ≤ 5), Single (0 < X ≤ 1), and NA).

Table S1 Primers used in H2K4me3 and H3K27me3 ChIP-qPCR.

| Primer name | Primer sequence (5’ to 3’) |
| --- | --- |
| *Tbx21*（-13605 bp） | F: AGGGACCGATCCTTGAGAA |
|  | R: GAGAGAAGGGCAAATCTCCA |
| *Tbx21*（+175 bp） | F: TTCCAGCAGCCGTCGAAG |
|  | R: TCCCGCTCCAGTGAAGTTTC |
| *Tbx21*（+262 bp） | F: CCCTCGTCACTCGGCATC |
|  | R: TTCGACGGCTGCTGGAAG |
| *Tbx21*（+5431 bp） | F: GAGGGACCAGGGACAGACT |
|  | R: CATCCCAGACCTAGCCACAG |
| *Hopx*（+2220 bp） | F: CCTGCCTCCGTATTGGAGACT |
|  | R: ACAGTTGTTTCCAGCCTTCCTC |
